# IL-27 Modulates Mesenchymal Stem Cell Immunoplasticity for Enhanced Lupus Nephritis Therapy via the JAK1–STAT1–IDO Axis and Tryptophan Metabolic Orchestration

**DOI:** 10.34133/research.0748

**Published:** 2025-07-10

**Authors:** Cheng Zhou, Shunlai Shang, Jing Zhao, Yunzhao Yang, Meihan Shi, Ping Li, Qinggang Li, Jian Zhang, Wenge Li, Chuyue Zhang, Xue-Yuan Bai

**Affiliations:** ^1^ Department of Nephrology, China-Japan Friendship Hospital, Beijing 100029, China.; ^2^ Department of Nephrology, First Medical Center of Chinese PLA General Hospital, State Key Laboratory of Kidney Diseases, National Clinical Research Center for Kidney Diseases, Beijing Key Laboratory of Medical Devices and Integrated Traditional Chinese and Western Drug Development for Severe Kidney Diseases, Beijing Key Laboratory of Digital Intelligent TCM for the Prevention and Treatment of Pan-Vascular Diseases, Key Disciplines of National Administration of Traditional Chinese Medicine (Zyyzdxk-2023310), Beijing 100853, China.; ^3^ Department of Nephrology and Institute of Kidney Diseases, West China Hospital, Sichuan University, Chengdu 610041, China.

## Abstract

Lupus nephritis is recognized as a common and severe complication of systemic lupus erythematosus, without an optimal therapeutic strategy currently available. While mesenchymal stem cells (MSCs) hold therapeutic promise, their efficacy varies substantially, likely due to their plasticity and capacity to adopt pro-inflammatory (MSC1) or anti-inflammatory (MSC2) functional states in response to different microenvironments. Here, we report for the first time that IL-27, via JAK1–STAT1 signaling, up-regulates indoleamine 2,3-dioxygenase (IDO) in MSCs, driving MSC differentiation toward an IDO-positive MSC2 phenotype with low immunogenicity. These IDO-positive MSC2 cells produce kynurenine and kynurenic acid, the metabolites of tryptophan, which bind to the intracellular aryl hydrocarbon receptor. This interaction stimulates an increase in the anti-inflammatory factor TSG-6 and induces the differentiation of regulatory T cells. Notably, IL-27-conditioned MSC2 demonstrated superior therapeutic efficacy compared to conventional MSCs in a murine lupus nephritis model. In conclusion, this study revealed that IL-27 is a critical modulator of MSC immune plasticity and presented a novel therapeutic strategy utilizing IL-27-enhanced MSC2 for autoimmune diseases.

## Introduction

Lupus nephritis (LN) is the most common and severe renal injury complication of systemic lupus erythematosus [1]. Current therapeutic approaches, including corticosteroids, immunosuppressants, and biologics, are only partially effective and often associated with marked side effects, leaving many patients without satisfactory treatment options [2,3]. This underscores the urgent need for novel and more effective therapeutic strategies for LN.

The immunoregulatory capabilities of mesenchymal stem cells (MSCs) position them as a prominent treatment strategy for managing autoimmune disorders [4,5]. However, their clinical efficacy has shown notable variability [6]. This inconsistency may be attributed to MSCs’ remarkable plasticity—their ability to adopt either pro-inflammatory (MSC1) or anti-inflammatory (MSC2) phenotypes depending on different microenvironments [7–11]. Understanding and controlling this plasticity is crucial for optimizing therapy based on MSCs.

The cytokine microenvironment plays a crucial role in regulating the phenotype of MSCs. Pro-inflammatory cytokines such as interferon-γ (IFN-γ) and tumor necrosis factor-α (TNF-α) can enhance the anti-inflammatory properties of MSCs by inducing the expression of immunosuppressive molecules, like indoleamine 2,3-dioxygenase (IDO) [12,13]. However, they also increase MSC immunogenicity, potentially limiting their therapeutic utility [14]. Beyond these well-studied cytokines, it remains unclear whether other cytokines can selectively enhance the anti-inflammatory properties of MSCs without increasing their immunogenicity.

IDO is a rate-limiting enzyme in tryptophan metabolism. By catalyzing the production of metabolites such as kynurenine (Kyn) and kynurenic acid (Kyna), it plays a central role in regulating immune responses [15]. These metabolites interact with the aryl hydrocarbon receptor (AHR) to modulate immune activity by promoting the production of regulatory T cells (Tregs) [16–18]. IDO expression is widely regarded as a marker of MSC anti-inflammatory capacity, yet the pathways regulating its expression and function in MSCs require further exploration [19–21].

IL-27 is a pleiotropic cytokine with marked anti-inflammatory and immunosuppressive properties [22–24]. Studies demonstrate its capacity to inhibit proliferation of pro-inflammatory T cells (Th1/Th17) while promoting Treg generation. This functional alignment with IDO’s mechanism stems from IDO-mediated catalysis of tryptophan into Kyns, which suppress T-cell activity and induce Treg differentiation, thereby attenuating excessive immune responses [25]. IL-27 has been demonstrated to directly regulate IDO expression in tumor cells while stimulating tryptophan metabolite production, establishing a dual immunometabolic regulatory pattern particularly prominent in inflammatory microenvironments [26,27]. Furthermore, as an endogenously expressed cytokine, IL-27 exhibits superior safety profiles compared to other pro-inflammatory factors (e.g., IFN-γ), with reduced risks of inflammatory responses and toxicity [28–30]. While IL-27 has not been directly confirmed to regulate IDO expression in MSCs, its intrinsic anti-inflammatory properties, synergistic mechanisms with IDO, metabolic regulatory superiority, and favorable safety profile warrant essential investigation into both the regulatory mechanisms of the MSC–IDO axis and their therapeutic applications.

Here, we reported for the first time that IL-27, via JAK1–STAT1 signaling, up-regulates IDO in MSCs, driving MSC differentiation toward an IDO-positive MSC2 phenotype with low immunogenicity. These MSC2 cells produce tryptophan metabolites that activate AHR, up-regulate the anti-inflammatory factor tumor necrosis factor stimulating gene 6 (TSG-6), and promote Treg differentiation. Using single-cell RNA sequencing (Sc-RNA-seq), we identified a distinct IDO+ MSC2 subpopulation induced by IL-27. In a murine LN model, IL-27-conditioned MSC2 exhibited superior therapeutic efficacy compared to conventional MSCs. This study not only uncovers IL-27 as a critical modulator of MSC immune plasticity but also provides a foundation for developing IL-27-enhanced MSC2 as an innovative therapeutic modality applicable to LN and other autoimmune disorders.

## Results

### IL-27 up-regulated the expression of anti-inflammatory molecule IDO by activating the JAK1–STAT1 signaling pathway in MSCs

**Fig. 1.**
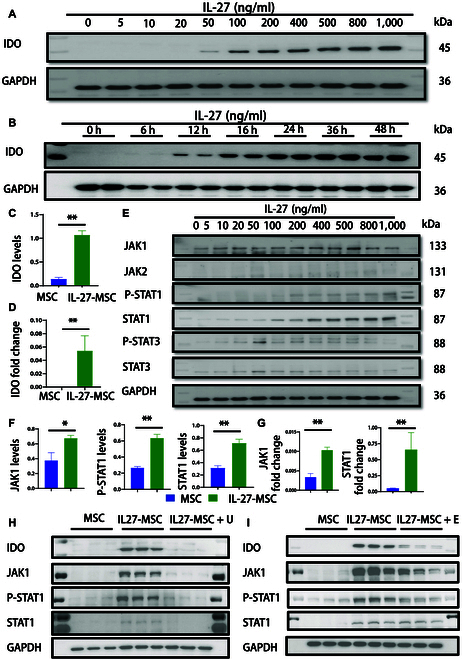
IL-27, via JAK1–STAT1 signaling, up-regulated the indoleamine 2,3-dioxygenase (IDO) level in mesenchymal stem cells (MSCs). (A) Protein expression level of IDO by Western blot in MSCs treated by different concentrations of IL-27. (B) Protein expression level of IDO by Western blot in MSCs treated for different time periods with stimulation by IL-27. (C and D) Histogram of the IDO expression level detected by Western blot and real-time quantitative polymerase chain reaction (qPCR) in MSCs treated by IL-27 at 200 ng/ml. (E) Protein expression of JAK-STAT by Western blot in MSCs treated by IL-27 at 200 ng/ml. (F) Histogram of JAK1–STAT1 expression detected by Western blot in MSCs treated by IL-27 at 200 ng/ml. (G) Histogram of JAK1–STAT1 expression detected by real-time qPCR in MSCs treated by IL-27 at 200 ng/ml. (H) Protein expression of the JAK1–STAT1–IDO pathway detected by Western blot in MSCs treated by IL-27 with a JAK1 inhibitor at 1 μM (U, upadacitinib). (I) Protein expression of the JAK1–STAT1–IDO pathway detected by Western blot in MSCs treated by IL-27 with an IDO inhibitor at 100 nM (E, epacadostat) (*P < 0.05; **P < 0.01).

IDO is a key enzyme involved in the immunosuppression of MSCs. The expression of IDO in MSCs could be induced by TNF-α and IFN-γ [19,31,32]. We found that IL-27 up-regulated IDO expression in MSC in a dose-dependent manner by Western blot and polymerase chain reaction (PCR) (Fig. 1A). Meanwhile, IL-27 (200 ng/ml) up-regulated IDO expression in MSCs in a time-dependent manner (Fig. 1B). After repeated experiments, 200 ng/ml and 24 h were selected as the processing concentration and time of IL-27, respectively, in the follow-up experiment (Fig. 1C and D).

To further verify the specificity of IDO expression in MSCs induced by IL-27, we also treated MSCs with IL-4/13/23/34 and IL-17A/B/C/D/E/F. Compared with IL-27, none of the above cytokines could effectively induce IDO overexpression in MSCs (Fig. S1A and B). Therefore, this finding indicated that IL-27 could specifically enhance the expression of IDO in MSCs.

To further elucidate the mechanism of IDO high expression in IL-27 priming, we measured the levels of JAK1/2, STAT1/2/3/4/5, and their phosphorylated forms in MSCs. The results showed that JAK1 and STAT1/P-STAT1 were increased in the IL-27-treated MSCs (IL-27-MSC), whereas JAK2 and STAT3/P-STAT3 did not change significantly (Fig. 1E to G). Additionally, STAT2/P-STAT2, STAT4/P-STAT4, and STAT5/P-STAT5 did not significantly alter (protein expression was low, and the results are not displayed). To clarify the JAK1–STAT1–IDO pathway in IL-27-MSC, we added a JAK1 inhibitor (upadacitinib) and an IDO inhibitor (epacadostat) in the induction system. We discovered that both JAK1 inhibitors and IDO inhibitors can inhibit the high expression of IDO, and JAK1 inhibitors can significantly inhibit the expression of JAK1 and STAT1/P-STAT1 (Fig. 1H and I).

### IL-27 priming retained the biological characteristics of MSCs without elevating their immunogenicity

**Fig. 2.**
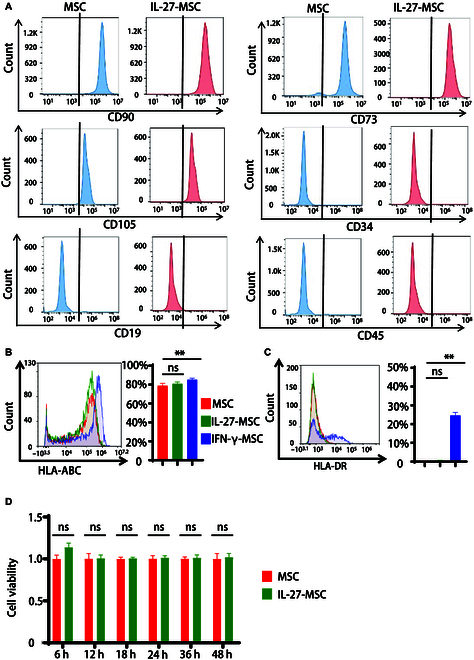
IL-27 priming retained the biological characteristics of conventional MSCs without elevating their immunogenicity. (A) Phenotype analyses of MSCs and IL-27-treated MSCs (IL-27-MSC) by flow cytometry. (B and C) Flow cytometry results of HLA-ABC and HLA-DR in IL-27-MSC and IFN-γ-MSC for 24 h (left) and histogram of the percentage of HLA-ABC+ cells and HLA-DR+ cells (right). (D) Detection of the proliferative ability of MSCs and IL-27-MSC by the Cell Counting Kit-8 (CCK-8) cell proliferation test. (*P < 0.05; **P < 0.01; ns, not significant).

The International Society of Cell Therapy formulated a basic definition of MSCs; that is, there is a high expression of CD90, CD73, and CD105, and CD34, CD19, CD45, and HLA-DR are not expressed. They could also differentiate into osteoblasts, adipocytes, and chondrocytes [33]. These markers in MSCs was detected by flow cytometry, and we found that MSCs still met the definition criteria of MSCs after IL-27 treatment (Fig. 2A and Fig. S1C).

To determine the advantages of IL-27 priming than IFN-γ priming, we evaluated the immunogenicity (MHC I molecule, HLA-ABC and MHC II molecules, and HLA-DR molecule) of MSCs. The results showed that IFN-γ-treated MSCs, which are currently employed to induce the anti-inflammatory MSC2, resulted in an increase in the levels of HLA-ABC and HLA-DR, whereas IL-27 treatment had no changes on HLA-ABC and HLA-DR in MSCs (Fig. 2B and C). We also discovered that IL-27 treatment had no effect on MSC proliferation by using Cell Counting Kit-8 proliferation tests (Fig. 2D).

### IL-27 priming MSCs strongly suppressed the activation and proliferation of T cells and fostered the generation of Tregs

**Fig. 3.**
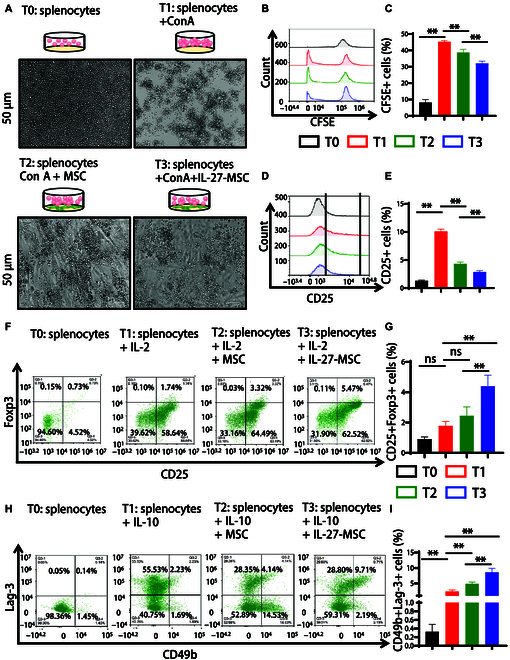
IL-27 enhanced MSC immunosuppressive function in inhibiting T cells’ proliferation and promoting regulatory-T-cell (Treg) differentiation in vitro. (A) The representative picture of splenic cell proliferation when the ratio of MSCs to splenic cell co-culture was 1:80. (B and C) Representative flow cytometry (left) and statistical histogram (right) of splenic cell proliferation by carboxyfluorescein succinimidyl ester (CFSE) assay in different groups. (D and E) Representative flow cytometry (left) and statistical histogram (right) of the T-cell activation marker CD25 in different groups. (F and G) Representative flow cytometry (left) and statistical histogram (right) of CD25+Foxp3+ Treg in different groups. (H and I) Representative flow cytometry (left) and statistical histogram (right) of CD49b+Lag-3+ Tr1 in different groups (*P < 0.05; **P < 0.01; ns, not significant). Con A, concanavalin A.

In order to look into how IL-27 affected MSC’s immunological function, we observed the MSC inhibitory effect on the activation and proliferation in mouse spleen cells. First, we extracted mouse spleen cells and induced their activation with concanavalin A (Con A). Then, these spleen cells were co-cultured with MSCs and IL-27-treated MSCs (IL-27-MSC) for 3 d. Adding Con A induced the spleen cells’ activation and made them aggregate into clusters, while IL-27-MSC could more effectively prevent splenic cell clumping than MSCs (Fig. 3A).

The fluorescent live cell dye carboxyfluorescein succinimidyl ester (CFSE) was then used to measure the proliferation of splenic cells. IL-27-MSC more effectively suppressed the proliferation of mice splenic cells than MSCs (Fig. 3B and C). Next, flow cytometry was used to detect the T-cell activation marker CD25. The results revealed that IL-27-MSC more markedly reduced CD25 expression in comparison to MSCs (Fig. 3D and E). To contrast the effects of untreated MSCs and IL-27-MSC on peripheral blood mononuclear cells’ activation and proliferation, MSCs were simultaneously co-cultured with human peripheral blood mononuclear cells. Compared to untreated MSCs, IL-27-MSC more effectively suppressed the proliferation and clumping of human peripheral T cells and the level of CD69 (early activation marker) and CD25 (late activation marker) on T cells (Fig. S2A to C).

Foxp3+ Tregs and Lag-3+CD49b+ Tregs (Tr1) are crucial for the immunosuppression function of MSCs. Our prior study revealed that MSCs could increase the percentage of Foxp3+ Tregs and Tr1 in the LN mouse kidneys [34]. Firstly, IL-2 was added to the culture medium to induce mouse spleen cell differentiation toward Foxp3+ Tregs. Then, they were co-cultured with MSCs and IL-27-MSC for 3 d. The results showed that IL-27-MSC more significantly increased the Foxp3+ Treg proportion compared to MSCs (Fig. 3F and G).

Then, IL-10 was added to the culture medium to induce mouse spleen cell differentiation toward Tr1 cells. Then, they were co-cultured with MSCs and IL-27-MSC for 3 d. The results showed that IL-27-MSC more significantly increased the Tr1 proportion compared to MSCs (Fig. 3H and I). Therefore, this study suggests that IL-27-MSC can more effectively promote the differentiation of 2 types of regulated T cells.

### IL-27 priming altered the expression of immune regulation genes in MSCs

**Fig. 4.**
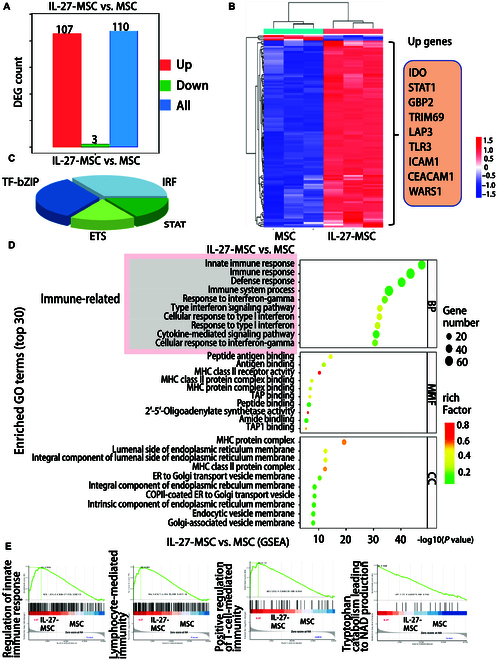
IL-27 priming altered the immune regulation genes in MSCs. (A) The number of differential genes between the MSC and IL-27-MSC groups by transcriptome analysis. (B) The differential genes between the MSC and IL-27-MSC groups. (C) The differential transcription factor analysis between the MSC and IL-27-MSC groups. (D) Gene Ontology (GO) enrichment analysis between the MSC and IL-27-MSC groups. (E) Gene set enrichment analysis (GSEA) between the MSC and IL-27-MSC groups. DEG, differentially expressed gene; BP, Biological Process; MF, Molecular Function; CC, Cellular Component; NES, normalized enrichment score; FDR, false discovery rate.

We analyzed the overall gene expression in MSCs and IL-27-MSC using RNA transcriptome sequencing to see how IL-27 affected the gene profile in 2 different MSCs. The results showed that IL-27-MSC underwent a considerable gene expression profile alteration. Compared to the MSC group, the IL-27-MSC group exhibited 110 differentially expressed genes (DEGs), of which 107 were up-regulated and 3 were down-regulated (Fig. 4A). The IDO and STAT1 gene expression levels were markedly elevated in IL-27-MSC (Fig. 4B). Immune-regulation-related genes, such as GBP2, TRIM69, LAP3, and TLR3, and cell adhesion-related genes, ICAM1 and CEACAM1, were up-regulated in IL-27-MSC.

In addition, in IL-27-MSC, the expression of the tryptophanyl-tRNA synthetase 1 (WARS1) gene involved in tryptophan metabolism was up-regulated. Furthermore, the differences in transcription factors between IL-27-MSC and MSCs mainly focused on 4 families: STAT, IRF, TF-bZIP, and ETS (Fig. 4C). Subsequently, we performed Gene Ontology (GO) enrichment analysis for differential genes. The results show that IL-27-MSC and MSCs have marked differences in immune-related pathways, like immune response, the immune system process, the cytokine-mediated signaling pathway, and the type I interferon signaling pathway (Fig. 4D). Gene set enrichment analysis revealed that IL-27 up-regulated immune-modulation-related pathways, like regulation of innate immune response, positive regulation of T-cell-mediated immunity, B-cell-mediated immunity, lymphocyte-mediated immunity, and the tryptophan metabolic pathway, like tryptophan catabolism leading to NAD production (Fig. 4E).

### IL-27 priming altered the landscape of the MSC subgroup and increased IDO+ stem cell clusters

Previous studies revealed that MSCs are a diverse, ill-defined, heterogeneous cell population that includes a number of subpopulations, such as proliferative, transition, stemlike, and functional subpopulations [35]. We analyzed the landscape of MSCs and IL-27-MSC by using 10× Genomics Sc-RNA-seq technology to gain insight into how IL-27 affected the differentiation of MSC subpopulations. In all, 25,931 cells were identified, 12,968 from the MSC group and 16,963 from the IL-27-MSC group.

**Fig. 5.**
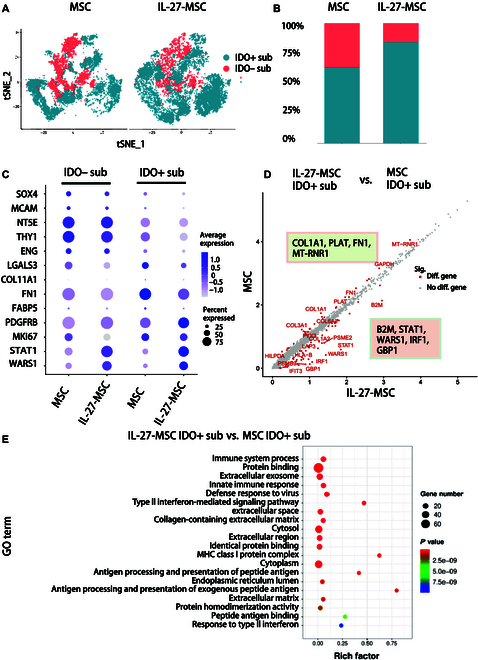
Single-cell RNA sequencing (Sc-RNA-seq) illustrated that IL-27 directed MSC differentiation toward an IDO-positive phenotype with up-regulated expression of stem cell differentiation genes. (A) The IDO+ subgroup (IDO+ sub) and IDO− subgroup (IDO− sub) on the *t*-distributed stochastic neighbor embedding (tSNE) plot; 12,968 cells from MSC and 16,963 cells from IL-27-MSC. (B) Proportions of IDO+ sub and IDO− sub among MSC and IL-27-MSC groups. (C) Dot plot for the relative expression levels of the DEGs in IDO+ sub and IDO− sub among the MSC and IL-27-MSC groups. The dot size indicates the percentage of cells in different clusters expressing the genes on the left; the color indicates the relative level of gene expression (high to low shown as blue to gray). (D) Scatter plot for the relative gene expression levels of the DEGs in IDO+ sub in IL-27-MSC vs. in IDO+ sub in MSCs. (E) GO enrichment analysis of IDO+ sub between the MSC and IL-27-MSC groups.

Based on the IDO gene expression level, the cells in both the MSC and IL-27-MSC groups were separately divided into the IDO+ subgroup (IDO+ sub) and IDO− subgroup (IDO− sub) (Fig. 5A). Compared with the MSC group, in the IL-27-MSC group, the proportion of IDO+ sub was increased (Fig. 5B). Analysis of differential genes in IDO+ sub and IDO− sub was then performed, and the results showed that in IDO+ sub, the genes related to stem cell differentiation (LGALS3, COL11A1, FN1, PDGFRB, and FABP5) and related to stem cell proliferation (MKI67) were up-regulated. In IDO− sub, the genes related to stem cell stemness (SOX4, MCAM, NT5E, THY1, and ENG) were up-regulated (Fig. 5C).

Next, we compared the differential genes between the IDO+ subgroup and the IDO− subgroup. The genes related to stem cell differentiation (LGALS3, COL11A1, FN1, PDGFRB, and FABP5) and stem cell proliferation (MKI67) were highly expressed in the IDO+ subgroup, and the genes related to stem cell stemness (SOX4, MCAM, NT5E, THY1, and ENG) were highly expressed in the IDO− subgroup (Fig. 5C). We then compared the differential genes of the IDO+ subgroup between the MSC and IL-27-MSC groups. In the IL-27-MSC group, the IDO+ subgroup showed higher expression levels of B2M, STAT1, WARS1, IRF1, and GBP1. In the MSC group, the IDO+ subgroup showed higher expression levels of COL1A1, PLAT, FN1, and MT-RNR1 (Fig. 5D). GO analysis of these differential genes showed that in the IL-27-MSC group, the IDO+ subgroup showed more substantial enrichment in the immune system process, innate immune response, and extracellular exosome pathways (Fig. 5E).

**Fig. 6.**
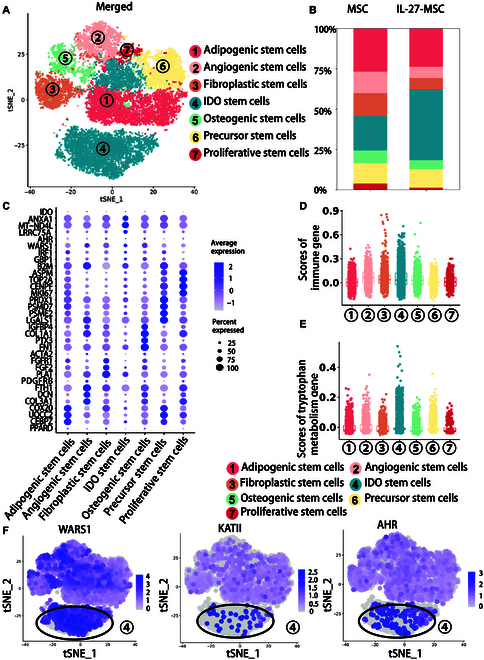
IL-27 priming induced highly IDO-positive clusters with anti-inflammatory capacity and enriched in tryptophan metabolism. (A) Cell cluster identification on the tSNE plot of 7 clusters in the merged groups (MSC and IL-27-MSC) of the IDO+ subgroup. (B) Proportions of 7 different clusters among the IDO+ subgroup in the MSC and IL-27-MSC groups. (C) Dot plot showing the DEGs’ relative expression levels in the 7 different clusters. The dot size indicates the percentage of cells in the 7 different clusters expressing the genes on the left; the color indicates the relative level of gene expression (high to low shown as blue to green). (D) The scores of the 7 different clusters for the immune regulation pathway. (E) The scores of the 7 different clusters for the tryptophan metabolism pathway. (F) tSNE plots of WARS1, KATII, and aryl hydrocarbon receptor (AHR) genes’ relative expression levels in the merged groups of the IDO+ subgroup.

Based on the specific up-regulated genes in the IDO+ subgroup, to identify which cell clusters had higher expression of IDO, we further defined them into 7 clusters: IDO+ stem cells, precursor stem cells, adipogenic stem cells, angiogenic stem cells, fibroblastic stem cells, osteogenic stem cells, and proliferative stem cells (Fig. 6A). The IL-27-MSC group had a greater percentage of IDO+ stem cells than the MSC group (Fig. 6B). In the IDO+ stem cell cluster, there was a high expression of IDO and immune-related genes (B2M, GBP1, IRF1, and ANXA1). Adipogenic stem cells were characterized with lipid-related genes (e.g., PPARD, CEBPZ, UQCC2, and COX20), fibroblastic stem cells were characterized with fibroblast-cell-related genes (e.g., PLAT, FGF2, FGFR1, and ACTA2), osteogenic stem cells were characterized with osteogenic genes (such as FN1, PTX3, COL1A1, and IGFBP4), proliferative stem cells were characterized with proliferation-related genes (e.g., MKI67, CENPF, TOP2A, and ASPM), and precursor stem cells were characterized with stem cell differentiation genes (such as LGALS1, PSME2, PSMD7, and PRDX1) (Fig. 6C).

We scored gene sets related to immunoregulation and tryptophan metabolism in the IDO+ stem cell cluster. Dataset analysis showed that IDO+ stem cells were active in immune regulation and tryptophan metabolism (Fig. 6D and E). In IDO+ stem cells, the expression levels of the tryptophan-metabolism-pathway-related genes, such as the tryptophan transporter gene (WARS1), key enzyme gene of catalytic tryptophan catabolism (KATII), and tryptophan metabolite AHR, were increased (Fig. 6F).

The pseudotime analysis of these 7 clusters revealed that the IDO+ stem cell cluster was positioned on the left branch of the differentiation trajectory, precursor stem cells occupied the upper branch of the trajectory, while the remaining clusters were located on the right side of the differentiation trajectory (Fig. S3A). The precursor stem cells at the starting points of differentiation trajectories had 2 possible differentiation fates: fate 1 (IDO+ stem cells) and fate 2 (osteoblastogenic/adipogenic/vascular/fibrogenic/proliferative stem cells) (Fig. S3B). Compared with the MSC group, the precursor stem cells differentiated toward IDO+ stem cells in the IL-27-MSC group were increased (Fig. S3C).

Therefore, we speculated that IL-27 treatment promoted the differentiation of IDO+ stem cells. Then, through transcription factor analysis, these transcription factors, AHR and ZEB1, were highly expressed in IDO+ stem cells (Fig. S3D and E). ZEB1 can reshape the local immunosuppressive microenvironment of tumors by regulating immune checkpoints CD47 and PD-1 (CD274) [36,37]. We found that in IDO+ stem cells, the expression levels of CD47 and CD274 increased (Fig. S3F). Thus, in a future study, CD47 and CD274 may be regarded as markers of IDO+ stem cells.

### IL-27 priming promoted tryptophan metabolism in MSCs and enhanced the level of the anti-inflammatory cytokine TSG-6

We have shown that IL-27 could increase IDO expression in MSCs. IDO, as a rate-limiting enzyme, regulates the metabolism of tryptophan. The main metabolic pathway of tryptophan in vivo is the Kyn metabolism pathway, which is related to immune response and inflammation. Tryptophan generates Kyn under the action of IDO. Kynurenine further generates Kyna under the action of Kyn aminotransferase (KATI to KATIV), which acts as a ligand to activate AHR [38–42].

**Fig. 7.**
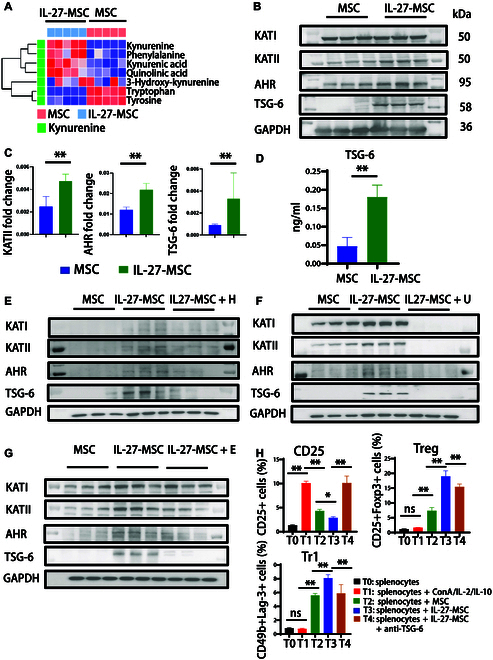
IL-27 priming MSCs produced kynurenine and kynurenic acid, the metabolites of tryptophan, and further enhanced the downstream expression of the cytokine tumor necrosis factor stimulating gene 6 (TSG-6). (A) Unsupervised clustering of tryptophan and 4 tryptophan metabolites in MSC and IL-27-MSC culture supernatant samples. (B) Western blot of the protein expression of KATI/KATII, AHR, and TSG-6 in MSC treated by IL-27 at 200 ng/ml. (C) Histogram of the Western blotting of protein expression and real-time qPCR of the RNA expression of KATII, AHR, and TSG-6 in MSC treated by IL-27 at 200 ng/ml. (D) Histogram of the enzyme-linked immunosorbent assay (ELISA) of the cytokine quantitative expression of TSG-6 in MSC treated by IL-27 at 200 ng/ml. (E) Western blot of the protein expression of the KAT–AHR–TSG-6 pathway in MSCs treated by IL-27 with a KAT inhibitor at 50 μM (H, homocysteine). (F) Western blot of protein expression of the KAT–AHR–TSG-6 pathway in MSCs treated by IL-27 with a JAK1 inhibitor at 1 μM (U, upadacitinib). (G) Western blotting of the protein expression of the KAT–AHR–TSG-6 pathway in MSCs treated by IL-27 with an IDO inhibitor at 100 nM (E, epacadostat). (H) Statistical histogram by flow cytometry of CD25+ cells, Tregs, and Tr1 cells in different groups. (*P < 0.05; **P < 0.01; ns, not significant).

Therefore, we subsequently employed liquid chromatography–mass spectrometry (LC–MS) to quantify tryptophan and its metabolic products, comparing the differences in their concentrations between the culture supernatants of MSCs and IL-27-MSC. There was a substantial difference in the distribution of tryptophan metabolites between the MSC and IL-27-MSC groups (Fig. 7A). In the MSC group, the contents of tryptophan and tyrosine were relatively high. The concentrations of Kyn and Kyna were higher in IL-27-MSC. The results of metabolite detection further indicated that IL-27 could promote the decomposition of tryptophan and produce its downstream metabolites, Kyn and Kyna.

The AHR signaling pathway serves as a critical regulatory mechanism in preserving immune system homeostasis. Kyn generates Kyna, an endogenous AHR activator, via Kyn aminotransferase (KATII). TSG-6, a downstream cytokine regulated by AHR, has strong anti-inflammatory properties [43]. As an effective inhibitor of neutrophil migration, it can inhibit inflammatory signals and contribute to the down-regulation of protease networks, which can be used as an indicator of the immunosuppressive ability of MSCs [44–46]. IL-27 also increased the expression levels of KATII, AHR, and TSG-6 genes and proteins in MSCs (Fig. 7B and C).

Using enzyme-linked immunosorbent assay (ELISA) technology, we found that the content of TSG-6 in the cell culture supernatant in the IL-27-MSC group was significantly higher than that in the MSC group (Fig. 7D). Then, we added a KATII inhibitor (homocysteine), a JAK1 inhibitor (upadacitinib), and an IDO inhibitor (epacadostat) into the above IL-27 induction system to observe whether the expression of AHR and TSG-6 could be blocked. The results showed that the KAT–AHR–TSG-6 pathway was inhibited after the addition of the above inhibitors (Fig. 7E to G). The ability of IL-27-MSC to inhibit T cells’ proliferation and activation and promote T cells’ differentiation into Tregs (Foxp3+ Tregs and Tr1) was also weakened after the addition of anti-TSG-6 antibody into the IL-27-MSC co-culture system with spleen cells (Fig. 7H and Fig. S4A to C).

### IL-27 priming enhanced the therapeutic effects of MSCs for LN in vivo

**Fig. 8.**
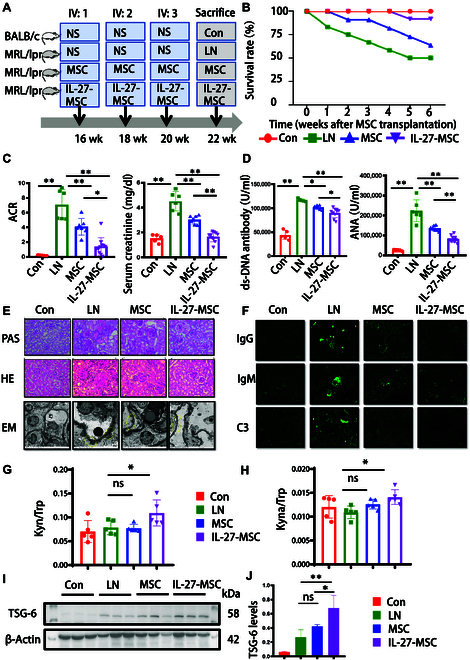
IL-27 priming MSCs demonstrated superior therapeutic efficacy compared to conventional MSCs in a murine lupus nephritis (LN) model via up-regulation of tryptophan metabolism and TSG-6 production. (A) Schematic of MSC and IL-27-induced MSC transplantation procedures. Mice were divided into 4 groups: the control group (Con), age-matched BALB/c mice (*n* = 5) receiving saline perfusion; the LN model group (LN), MRL/lpr mice (*n* = 11) receiving saline perfusion; the MSC treatment group (MSC): MRL/lpr mice (*n* = 11) receiving MSC (1 × 10^6^ cells) perfusion; and the IL-27-induced MSC treatment group (IL-27-MSC), MRL/lpr mice (*n* = 11) receiving IL-27-MSC (1 × 10^6^ cells) perfusion. (B) Survival rates of the 4 groups from 0 to 6 weeks post-MSC transplantation. (C) Urinary albumin-to-creatinine ratio (ACR) and serum creatinine levels in the 4 groups at 6 weeks posttransplantation. (D) Serum concentrations of anti-double-stranded DNA (anti-dsDNA) antibodies and anti-nuclear antibodies (ANAs) in the 4 groups. (E) Representative periodic acid–Schiff (PAS) staining, hematoxylin–eosin (HE) staining, and electron microscopy (EM) images of the 4 groups. Scale bars: ×400 = 25 μm and ×8,000 = 2 mm. (F) Representative images of IgM, IgG, and C3 deposition in kidneys across groups. (G) Kynurenine/tryptophan (Kyn/Trp) and (H) kynurenine acid/tryptophan ratios (Kyna/Trp) in MSC and IL-27-MSC serum samples. (I) Western blot of the protein expression of the TSG-6 pathway in the above 4 groups. (J) Histogram of TSG-6 in the above 4 groups (data presented as mean ± standard error of the mean [SEM]; *P < 0.05; **P < 0.01). EM,

To further clarify the therapeutic effect of IL-27-MSC in vivo, we observed the changes in IL-27-MSC on survival and renal function of LN model mice. LN model mice were given caudal intravenous injections of saline, MSCs, and IL-27-MSC at 16 weeks of age, followed by another injection at 18 and 20 weeks and killed at 22 weeks (6 weeks after treatment) (Fig. 8A). After 6 weeks of treatment, the survival rate in the LN model group (normal saline [NS]) was 54%, that in the MSC treatment group was 64%, and that in the IL-27-MSC treatment group was 91%, indicating that IL-27-MSC treatment prolonged the LN mice survival time (Fig. 8B and Fig. S5A). The levels of urinary albumin/creatinine ratio, serum creatinine, and serum anti-double-stranded DNA antibodies and anti-nuclear antibodies were all decreased in the MSC and IL-27-MSC groups compared with those in LN model group mice. Compared with MSC, IL-27-MSC treatment can more significantly reduce the above indexes (Fig. 8C and D).

Hematoxylin–eosin and periodic acid–Schiff staining of renal tissue showed that after 6 weeks of treatment, compared with that in the LN model group, glomerular cell proliferation in the MSC group and IL-27-MSC group was weakened, and the mesangial matrix was significantly reduced. Inflammatory cells were significantly reduced, and inflammatory cells appeared only occasionally. Compared with those in the MSC group, the pathological damages of the kidneys in the IL-27-MSC group were less severe, indicating that IL-27-MSC treatment could more significantly reduce the pathological damages of the kidneys in mice (Fig. 8E and Fig. S5B). Electron microscopy analysis also showed that after 6 weeks of treatment, more electron-dense matter was deposited under the glomerular epithelium or endothelial cells, and the foot process fusion was obvious in the LN model group. Electron-dense deposition and foot process fusion were less in the MSC group than in the LN group. The deposition of electron-dense matter and foot fusion in the IL-27-MSC group were significantly reduced compared with those in the LN model group and MSC group (Fig. 8E and Fig. S5C).

The main immunopathological changes in LN renal tissue were depositions of IgM, IgG, C3, and immune complex. Immunofluorescence staining results showed that IgM, IgG, and C3 depositions could not be seen in the glomeruli of normal mice (Con group). The glomeruli’s IgM, IgG, and C3 deposition in the LN group was obvious. The deposition in the glomeruli of the MSC and IL-27-MSC groups was significantly decreased compared with that of the LN group. Compared with that in MSCs, the deposition in renal tissue in the IL-27-MSC group was more significantly decreased (Fig. 8F and Fig. S5D).

The serum levels of tryptophan and its derivatives were compared by liquid mass spectrometry. We found that the ratios of the downstream metabolites of tryptophan, kynurenine/tryptophan (Kyn/Trp) and kynurenic acid/tryptophan (Kyna/Trp), were higher in the IL-27-MSC group (Fig. 8G and H).

Kyna can activate the expression of the anti-inflammatory cytokine TSG-6. We subsequently examined the level of TSG-6 in each group of mice’s kidney tissues. We discovered that the TSG-6 expression level was increased in the kidneys of mice in the IL-27-MSC group relative to that in the untreated MSC group (Fig. 8I and J). Then, we used antibody microarrays to analyze the changes in cytokine expression in the kidneys of LN mice after MSC and IL-27-MSC treatment. IL-10 and IL-2 were up-regulated after IL-27-MSC treatment (Fig. S5E and F). IL-2 and IL-10 may be crucial in Treg differentiation induced by IL-27-MSC.

## Discussion

MSCs have shown potential therapeutic value in immune-related diseases due to their immunomodulatory plasticity. However, their clinical application still faces 3 major scientific challenges: the uncertainty in biological characteristics caused by heterogeneous cell populations, the biphasic nature of immunomodulatory functions mediated by microenvironment-dependent phenotypic plasticity (pro-inflammatory/anti-inflammatory phenotype), and heterogeneity in efficacy in the treatment of LN, which has been shown by preclinical studies. Basic research has confirmed that naive MSCs need external stimulation to form a functional anti-inflammatory phenotype (MSC2), but traditional inducers such as IFN-γ or TNF-α may pose an immunogenic risk. This study found that IL-27 up-regulates the expression of the key enzyme IDO in tryptophan metabolism, maintaining the low immunogenicity characteristics of MSCs while achieving phenotypic stabilization. Compared with traditional induction schemes, the IDO+ immunosuppressive subpopulation induced by IL-27 has more stable immunosuppressive efficacy in vitro and in vivo. This discovery provides a new target for optimizing the precise regulation strategy of MSC treatment for LN.

The metabolism of tryptophan regulates a wide range of physiological and pathological processes, such as immune responses, growth regulation, metabolism, mood [47]. The core role of tryptophan metabolism regulation in the immunomodulatory mechanism of MSCs has achieved important research progress. MSCs dominate the tryptophan metabolic network through the high expression of IDO, which catalyzes tryptophan decomposition via the Kyn pathway into Kyn. This mechanism achieves immunosuppression through the dual mechanisms of microenvironmental tryptophan depletion and generation of immunomodulatory metabolites. Experimental data show that IFN-γ can increase IDO expression in MSCs. By promoting the expansion of Tregs and inhibiting Th17 differentiation, it exhibits therapeutic effects in rheumatoid arthritis models [48]. Metabolic reprogramming strategies such as supplementing tryptophan analogs can enhance the antioxidant stress capacity of MSCs and show effects in improving mitochondrial function in MSC senescence [49]. IL-27 can induce the high expression of IDO in tumor cells, which is related to tumor immune escape. Tryptophan depletion and production of Kyn and other metabolites could lead to immunosuppressive effects in the tumor microenvironment [50]. Combined with tryptophan metabolomics, this study innovatively reveals that IL-27 has the ability to regulate the tryptophan metabolism of MSCs, enhance the immunosuppressive capacity of MSCs, and induce MSCs to transform into anti-inflammatory type MSCs. At the same time, single-cell sequencing analysis reveals the IDO+ stem cell subpopulation with tryptophan metabolic advantages. This subpopulation enhances the immunomodulatory ability of MSCs by strengthening the Kyn pathway, establishing a theoretical foundation for more precise examination of the metabolic features of the anti-inflammatory subtype of MSCs.

As shown in Results, IL-27 up-regulated IDO in MSCs through the JAK1–STAT1 signaling axis. IL-27-treated MSCs exhibited increased JAK1 and STAT1 phosphorylation, with no changes observed in JAK2 or STAT3. IL-27 (IL-12 family) binds its receptor complex (WSX-1/gp130) to activate JAK/STAT [51]. While typically activating both STAT1 and STAT3 in T cells to regulate Th1/Treg differentiation, IL-27 preferentially engages JAK1–STAT1 in MSCs, reflecting a cell-type-specific signaling pathway. In MSCs, STAT1 shows a higher affinity for the IDO promoter, while STAT3 regulates other genes. JAK1’s preferential receptor binding underlies this selectivity. The JAK2-STAT3 axis critically mediates stem cell osteogenesis [52]. Imbalanced STAT signaling contributes to therapeutic heterogeneity: STAT3 hyperactivation increases oncogenic risk, while STAT5 activity regulates the osteoblastic differentiation program in mesenchymal cells [53,54]. The distinct roles of JAK-STAT pathways in MSCs remain incompletely elucidated. Their cross talk with metabolic pathways (e.g., mTOR/AMPK) may complexly influence IDO-mediated tryptophan metabolism, warranting further investigation.

The transcription factor AHR is ligand activated and regulates both innate and adaptive immune responses. According to recent research on tryptophan metabolites, Kyna, which is generated by the metabolism of Kyn, is one of the ligands to AHR. The ligand–AHR complex can enter the nucleus after binding, thus regulating the expression of genes downstream. TSG-6 may be one of the downstream target genes of AHR in MSCs, according to our study. We observed that elevated IDO expression in IL-27-MSC promoted the generation of tryptophan metabolites (Kyn and Kyna), triggered the expression of AHR, and stimulated the expression of the downstream gene TSG-6. Meanwhile, we discovered that the genes related to tryptophan metabolism and the TSG-6 pathway were up-regulated in IDO+ stem cells.

One of the subgroups of anti-inflammatory MSC2 may be IDO+ stem cells. IL-27 could promote the development of these cluster cells. Potential markers (CD47 and CD274) of these cluster cells were hypothesized by single-cell RNA sequencing; however, they needed more investigation and validation. In subsequent research, we plan to sort these cluster cells in MSCs and then investigate their function in vitro and vivo.

The amino acid sequence of TSG-6 is highly conserved, with around 94% identity between humans and mice. TSG-6 possesses anti-inflammatory characteristics, such as blocking neutrophil migration, reducing inflammatory signals, and helping to down-regulate protease networks in renal inflammation [55]. In response to inflammatory signals, MSC may produce TSG-6, which mediates a variety of immunomodulatory and repair processes. According to this study, IL-27 stimulated MSCs to produce TSG-6, prevented T-cell activation and proliferation, and fostered the generation of Tregs. Additionally, we found that the kidney tissue of LN mice administered with IL-27-MSC had higher levels of TSG-6. However, the study’s weakness is the lack of in-depth investigation into the mechanism of how TSG-6 modulates LN immune response in the renal region. In future investigations, we will investigate the regulation effect of TSG-6 released by MSCs on LN renal immune cells, as well as the repair effect of kidney tissue damage.

IL-27-MSC demonstrated marked efficacy in LN by inducing an IDO+ anti-inflammatory phenotype and activating the tryptophan metabolism–AHR–TSG-6 axis. This mechanism may extend to other autoimmune diseases, including rheumatoid arthritis and multiple sclerosis. In rheumatoid arthritis, IL-27-MSC may alleviate joint inflammation by suppressing Th17 activation and promoting Treg expansion, while TSG-6 protects articular cartilage through inhibition of hyaluronan degradation [56]. In multiple sclerosis, they may modulate the central nervous system immune microenvironment to attenuate demyelination [57]. In type 1 diabetes, IL-27-MSC may activate IDO to expand Tregs in pancreatic lymph nodes, thereby inhibiting β-cell destruction by effector T cells, with TSG-6 potentially mitigating islet inflammation [58]. In systemic sclerosis, IL-27-MSC likely suppress TGF-β signaling through TSG-6, reducing collagen deposition and cutaneous fibrosis while improving vascular dysfunction and Raynaud’s phenomenon [59].

**Fig. 9.**
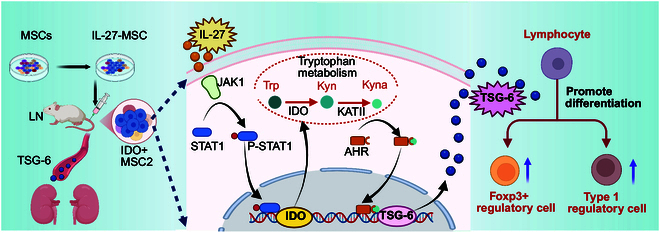
The overview diagram of IDO+ stem cell induction by IL-27 and its application in LN mouse. This diagram was created with BioRender.com with license. Trp, tryptophan; Kyn, kynurenine; Kyna, kynurenic acid.

In conclusion, this study for the first time discovered that IL-27 could increase the production of Kyn and Kyna in MSCS by activating the JAK1–STAT1 signaling pathway, promote MSC transformation into anti-inflammatory MSC2, increase the population of IDO+ stem cells, and enhance MSC immune regulation function (Fig. 9); Kyna increased the production of the anti-inflammatory factor TSG-6 by binding with AHR and improved the effectiveness of MSCS in LN mice. This finding provides a theoretical foundation for the potential application of IL-27-MSC in the treatment of LN and other immunological disorders.

## Materials and Methods

### MSC preparation and induction

The preparation of all umbilical cord MSCs was performed in accordance with the standardized operating procedures of the Stem Cell Research Center, and they were obtained from Qingdao Hualing Aoyuan Bioengineering Technology. We used the fourth-generation MSCs for experiments and treatment. Cells were cultured in Mesenchymal Stem Cell Medium (Cat. No. 7501, ScienCell, USA) in a humidified incubator at 37 °C with 5% CO_2_. Cells were treated by IL-27 in different concentrations for different time periods when cells were more than 70% confluent.

### Spleen cells’ isolation and co-culture with MSCs

The mouse spleen cell isolation protocol was performed as described previously. A Transwell chamber system (polycarbonate membrane, 0.4-μm pores, Corning) facilitated the co-culture microenvironment. MSCs were quantified and plated in lower compartments after receiving 10-Gy irradiation to inhibit cellular proliferation. Subsequently, the isolated splenocytes were enumerated and maintained in upper chambers at a 1:80 MSC-to-splenocyte ratio. The culture medium consisted of RPMI-1640 supplemented with 10% fetal bovine serum (Gibco), 2 mM l-glutamine, nonessential amino acid solution (1% v/v, Sigma), and essential amino acids. Pharmacological modulators (agonists/antagonists) were introduced into this co-culture environment according to experimental requirements.

### Spleen cells’ proliferation assay

Before MSC-to-splenocyte co-culture, MSCs were pretreated with or without IL-27 (200 ng/ml) for 24 h. After a 72-h co-culture with MSCs or IL-27-MSC, splenocytes were harvested. These splenocytes were placed on a 96-well microplate and treated with Con A, 10 mg/ml) for T-cell activation for 24 h. Spleen cells were harvested to examine the change in fluorescence intensity using CFSE (BioLegend, San Diego, CA, USA) proliferation assay. The higher-fluorescence cells could be identified as the parent generation.

### Flow cytometry

Flow cytometry was conducted as previously described. Briefly, spleen-derived cells and MSCs were collected and subjected to 2 centrifugation steps in phosphate-buffered saline (PBS). Cell suspensions were then treated with fluorochrome-labeled specific antibodies or corresponding isotype controls (BD) for 30 min under light-protected conditions. After dual washing cycles with cold buffer, cellular samples were acquired using flow cytometers (BD). Subsequent data interpretation was performed utilizing the FlowJo software.

### MSC identification and proliferation assay

The following antibodies for MSC identification were purchased from BioLegend: CD90, CD105, CD73, CD34, CD19, CD45, HLA-ABC, and HLA-DR for flow cytometry. WST-8 cell proliferation kits (Dojindo Molecular Technologies, Shanghai, China) were used to assess the proliferation of MSCs.

### Induction of Tregs (Foxp3+ Tregs and Lag-3+CD49b+ Tregs)

IL-2 (5 ng/ml, PeproTech) was added to the culture system for Foxp3+ Treg induction; 10 ng/ml IL-10 and IL-2 (PeproTech) were added to the culture system for Lag-3+CD49b+ Treg (Tr1) induction. Before induction, all T cells were activated by CD3/CD28 antibodies (BD Biosciences). The following antibodies for Treg identification were purchased from BioLegend: CD25, Foxp3, Lag-3, and CD49b. The data were analyzed by the FlowJo V software.

### Western blot

After IL-27 induction, MSCs were washed with PBS twice and digested by pancreatic enzymes. Total cellular proteins were isolated using radioimmunoprecipitation assay extraction buffer combined with 1 mM phenylmethanesulfonylfluoride proteinase inhibitor cocktail (Beyotime, China) and phosphatase inhibitor cocktail (Yeasen, China). Then, protein concentration determination was performed using BCA Protein Assay Kit (Beyotime). Western blotting was conducted according to a prior protocol. Protein samples (50 μg) were separated on a polyacrylamide–sodium dodecyl sulfate gel (10×) and electroblotted onto a nitrocellulose membrane. Then, the nitrocellulose membranes were placed in a 5% bovine serum albumin solution to block nonspecific protein for 2 h at room temperature; the membranes were incubated with specific antibodies, such as JAK1, JAK2, STAT1 to STAT5, P-STAT1 to P-STAT5, IDO, KATI, KATII, AHR, and TSG-6 (Abcam), overnight at 4 °C. The second day, the membranes were incubated with horseradish peroxidase-conjugated secondary antibodies (Beyotime, Shanghai, China) and then visualized using enhanced chemiluminescence.

### Real-time PCR

After IL-27 induction, MSCs underwent 2 PBS rinses for purification. Cellular RNA isolation was conducted using the TRIzol reagent (Thermo Fisher Scientific, USA) following the manufacturer’s protocol, with subsequent quantification performed via NanoDrop 2000 spectrophotometry (Thermo Fisher Scientific). For complementary DNA (cDNA) synthesis, 1 μg of total RNA was processed using PrimeScript RT Reagent Kit (Takara Bio, Japan). Quantitative PCR amplification was carried out in 10-μl reactions containing SYBR Select Master Mix (Applied Biosystems) on a QuantStudio 3 Real-Time PCR system, with thermal cycling parameters set according to primer specifications. GAPDH expression served as the internal normalization standard.

### Enzyme-linked immunosorbent assay

The concentrations of TSG-6 in MSC culture supernatant were measured by ELISA (R&D Systems, Minneapolis, MN) according to the manufacturer’s instructions.

### Sample and library preparation for MSC 10× single-cell sequencing

The cultured MSCs and IL-27-pretreated MSCs were digested with 0.25% trypsin for 3 min, then suspended in PBS to form cell pellets, and filtered through sterile 40-μm cell strainers. Using an automated fluorescence cell counter and trypan blue staining, cell viability was determined to be >80%. The MSC single-cell suspension was loaded onto a microwell chip, and bar-coded magnetic beads from the microwell chip were collected to capture messenger RNA labeled with bar-coded magnetic beads for reverse transcription to obtain cDNA. After quality assessment of cDNA (using a bioanalyzer), paired-end sequencing (150 bp) was performed on the Illumina NovaSeq 6000 platform, with a sequencing depth of at least 100,000 reads per cell. The single-cell RNA sequencing using the 10× Genomics Chromium platform and technical services were provided by LC-Bio Technology Co., Ltd. (Hangzhou, China).

### Raw data processing and preliminary analysis of single-cell sequencing

Using Cell Ranger, raw sequencing data underwent base calling, adapter trimming, and demultiplexing to obtain gene expression matrices for sequenced samples. Subsequently, the R software was employed to filter out low-quality cells with >6,000 or <301 expressed genes or >30% of unique molecular identifiers originating from mitochondrial genomes. Data dimensionality reduction and clustering were performed using functions in Seurat v3.1.2, with gene normalization and scaling (NormalizeData and ScaleData functions). The top 30 principal components were selected for downstream analysis and visualization. The final dataset comprised 25,931 cells, including 12,968 cells from the MSC group and 16,963 cells from the IL-27-MSC group.

### Differential gene expression and cell type analysis

Using the FindAllMarkers function in Seurat, marker genes within each cluster were determined via the Wilcoxon rank-sum test. Genes with expression exceeding 10% in a specific cluster and an average log(fold change) value greater than 0.25 were selected as DEGs. We integrated the FindAllMarkers function (test.use = presto) in Seurat to identify marker genes for each cluster and manually annotated cell clusters by correlating them with marker genes from prior studies on MSC differentiation subpopulations. Heatmaps, dot plots, and violin plots illustrating the expression of cell-type-specific markers were generated using the DoHeatmap, DotPlot, and VlnPlot functions in Seurat v3.1.2, respectively.

### Pathway and functional enrichment analysis

Based on the expression of DEGs, we performed GO and Kyoto Encyclopedia of Genes and Genome (KEGG) pathway enrichment analyses using the clusterProfiler R package [55]. GO terms and KEGG pathways with adjusted *P* values <0.05 were considered significantly enriched. Gene sets were downloaded from Mouse Genome Informatics (http://www.informatics.jax.org/). To further clarify the up-regulation or down-regulation of specific pathways and functions across different MSC groups, gene set enrichment analysis was performed using the clusterProfiler package in R. Genes were ranked based on their log_2_ fold change values, and the hallmark gene sets (MSigDB v2023.1) were evaluated. Significance thresholds were set at a normalized enrichment score absolute value >1.0 and false discovery rate *q* value <0.25. Gene sets containing at least 15 genes and a maximum of 500 genes were included in the analysis to avoid overfitting small or large sets.

### Pseudotime trajectory analysis of differentiation

The pseudotime cell differentiation trajectories of MSCs and IL-27-MSC were reconstructed using the Monocle 2 package. Cells were classified according to spatiotemporal differentiation order using DEGs. Dimensionality reduction and cell ordering along the trajectory were performed using the DDRTree method, and the differentiation trajectory was visualized using the plot_cell_trajectory function.

### Transcription factor analysis

Transcriptional regulatory network analysis was performed using SCENIC. Based on the distribution specificity of regulatory elements across cell subpopulations, the top transcriptional factor regulators with high regulator specificity scores ) were quantified. According to the description of LC-Bio Technology Cloud Platform, we conducted the aforementioned single-cell sequencing data analysis.

### LC–MS-analysis-based targeted metabolomic analysis

The culture supernatant was collected after termination of cultivation and filtered through 0.22-μm membranes to remove cellular debris. Metabolites were enriched using Oasis HLB solid-phase extraction columns (Waters). After activation steps (methanol–0.1% ammonia water), acidified samples (adjusted to pH 3.0 with 0.1 M HCl) were loaded. The eluent consisted of methanol/0.1% ammonia water (80:20, v/v). Chromatographic separation was performed on a Waters HSS T3 column (2.1 × 100 mm, 1.8 μm) with mobile phases of 0.1% formic acid in water (A) and acetonitrile (B). The gradient elution program was as follows: 0 to 2 min holding 5% B, 2 to 8 min increasing to 30% B, and 8 to 10 min reaching 70% B, at a flow rate of 0.4 ml/min. Mass spectrometry detection employed a Sciex 6500+ system in ESI+ mode, using multiple-reaction monitoring to monitor metabolites such as tryptophan (*m*/*z* 205.1 → 188.1) and Kyn (*m*/*z* 209.1 → 94.0). Method validation demonstrated linearity over 0.1 to 500 ng/ml (*R*^2^ > 0.99), interday precision relative standard deviation <12.5%. This protocol enables assessment of MSC-secreted tryptophan metabolites (e.g., Kyn/Trp ratio) and their immunomodulatory functions, providing metabolomic insights for mechanistic studies of stem cell therapies.

### Animals

The animal experiments in this study were conducted in the approved experimental protocol by the Medical Ethics Committee of the Chinese People’s Liberation Army General Hospital (Ethic Approval No. SQ2021209). Female MRL/lpr mice obtained from Shanghai SLAC Laboratory Animal Company Limited (Shanghai, China) and BALB/c mice obtained from Beijing Vital River Laboratory Animal Technology were used in this study. These mice were housed in the Medical Experimental Animal Center of the Chinese People’s Liberation Army General Hospital in a specific-pathogen-free environment with a 12-h light–dark cycle, at 24 to 26 °C temperature.

### Transplantation protocol of MSCs

Sixteen-week-old mice were randomized into 4 groups: (a) the control group, age-matched BALB/c mice (*n* = 5); (b) the LN group, MRL/lpr mice injected with 1 ml of NS via the caudal vein (*n* = 11); (c) the MSC group, MRL/lpr mice injected with MSCs (1 × 10^6^ cells, 1 ml) via the caudal vein (*n* = 11); and (d) the IL-27-MSC group, MRL/lpr mice injected with IL-27-MSC (1 × 10^6^ cells, 1 ml) via the caudal vein (*n* = 11). The mice in each group received intravenous administration of NS, MSC, or IL-27-MSC at 16, 18, and 20 weeks of age. Random urine samples were taken every 2 weeks. At 22 weeks of age, 24-h urine samples were taken and mice were anesthetized and sacrificed. Blood and kidney samples were collected and used by molecular biology experiments to evaluate the therapeutic effect of MSC treatment.

### Analysis of serum and urine samples and kidney histopathology

The levels of urinary albumin/creatinine ratio, serum creatinine, and serum anti-double-stranded DNA, and anti-nuclear antibodies were measured as described previously. Renal specimens obtained from murine models were postfixed with 4% paraformaldehyde for 48 h prior to processing. The tissues underwent sequential dehydration through a graded ethanol series, followed by paraffin embedding and preparation of ultrathin sections (2-μm thickness). Histopathological evaluation was performed through hematoxylin–eosin staining, periodic acid–Schiff reaction, and immunofluorescence microscopy targeting immunoglobulins (IgG and IgM) and complement component C3, following established protocols for renal pathology assessment.

### Statistics

Statistical analyses were performed using the GraphPad Prism software (version 8.0.1). Continuous variables are reported as mean ± SD. The Student *t* test was applied for 2-group comparisons, while one-way analysis of variance was employed for multigroup analyses. Significance levels are denoted as follows: *P < 0.05 (significant), **P < 0.01 (highly significant), and ns (not significant).

## Data Availability

The datasets utilized in this research are accessible through correspondence with the primary investigator following a reasonable request.

## References

[1] Siegel CH, Sammaritano LR. Systemic lupus erythematosus: A review. JAMA. 2024;331(17):1480–1491. 38587826 10.1001/jama.2024.2315

[2] Anders HJ, Saxena R, Zhao MH, Parodis I, Salmon JE, Mohan C. Lupus nephritis. Nat Rev Dis Primers. 2020;6(1):7. 31974366 10.1038/s41572-019-0141-9

[3] Lo MS, Tsokos GC. Treatment of systemic lupus erythematosus: New advances in targeted therapy. Ann N Y Acad Sci. 2012;1247:138–152. 22236448 10.1111/j.1749-6632.2011.06263.x

[4] Li A, Guo F, Pan Q, Chen S, Chen J, Liu HF, Pan Q. Mesenchymal stem cell therapy: Hope for patients with systemic lupus erythematosus. Front Immunol. 2021;12: Article 728190. 34659214 10.3389/fimmu.2021.728190 PMC8516390

[5] Li W, Chen W, Sun L. An update for mesenchymal stem cell therapy in lupus nephritis. Kidney Dis. 2021;7(2):79–89. 10.1159/000513741 PMC8010225 33824866

[6] Deng D, Zhang P, Guo Y, Lim TO. A randomised double-blind, placebo-controlled trial of allogeneic umbilical cord-derived mesenchymal stem cell for lupus nephritis. Ann Rheum Dis. 2017;76(8):1436–1439. 28478399 10.1136/annrheumdis-2017-211073

[7] Kozlowska U, Krawczenko A, Futoma K, Jurek T, Rorat M, Patrzalek D, Klimczak A. Similarities and differences between mesenchymal stem/progenitor cells derived from various human tissues. World J Stem Cells. 2019;11(6):347–374. 31293717 10.4252/wjsc.v11.i6.347 PMC6600850

[8] Ullah I, Subbarao RB, Rho GJ. Human mesenchymal stem cells—Current trends and future prospective. Biosci Rep. 2015;35(2): Article e00191. 25797907 10.1042/BSR20150025 PMC4413017

[9] Bernardo ME, Fibbe WE. Mesenchymal stromal cells: Sensors and switchers of inflammation. Cell Stem Cell. 2013;13(4):392–402. 24094322 10.1016/j.stem.2013.09.006

[10] Li Y, Jin M, Guo D, Shen S, Lu K, Pan R, Sun L, Zhang H, Shao J, Pan G. Unveiling the immunogenicity of allogeneic mesenchymal stromal cells: Challenges and strategies for enhanced therapeutic efficacy. Biomed Pharmacother. 2024;180: Article 117537. 39405918 10.1016/j.biopha.2024.117537

[11] Tfilin M, Gobshtis N, Fozailoff D, Fraifeld VE, Turgeman G. Polarized anti-inflammatory mesenchymal stem cells increase hippocampal neurogenesis and improve cognitive function in aged mice. Int J Mol Sci. 2023;24(5):4490. 36901920 10.3390/ijms24054490 PMC10003244

[12] DelaRosa O, Lombardo E, Beraza A, Mancheño-Corvo P, Ramirez C, Menta R, Rico L, Camarillo E, García L, Abad JL, et al. Requirement of IFN-γ–mediated indoleamine 2,3-dioxygenase expression in the modulation of lymphocyte proliferation by human adipose–derived stem cells. Tissue Eng Part A. 2009;15(10):2795–2806. 19231921 10.1089/ten.TEA.2008.0630

[13] Shi Y, Wang Y, Li Q, Liu K, Hou J, Shao C, Wang Y. Immunoregulatory mechanisms of mesenchymal stem and stromal cells in inflammatory diseases. Nat Rev Nephrol. 2018;14(8):493–507. 29895977 10.1038/s41581-018-0023-5

[14] Galipeau J, Sensébé L. Mesenchymal stromal cells: Clinical challenges and therapeutic opportunities. Cell Stem Cell. 2018;22(6):824–833. 29859173 10.1016/j.stem.2018.05.004 PMC6434696

[15] Vécsei L, Szalárdy L, Fülöp F, Toldi J. Kynurenines in the CNS: Recent advances and new questions. Nat Rev Drug Discov. 2013;12(1):64–82. 23237916 10.1038/nrd3793

[16] Fujiwara Y, Kato S, Nesline MK, Conroy JM, DePietro P, Pabla S, Kurzrock R. Indoleamine 2,3-dioxygenase (IDO) inhibitors and cancer immunotherapy. Cancer Treat Rev. 2022;110: Article 102461. 36058143 10.1016/j.ctrv.2022.102461 PMC12187009

[17] Stone TW, Williams RO. Modulation of T cells by tryptophan metabolites in the kynurenine pathway. Trends Pharmacol Sci. 2023;44(7):442–456. 37248103 10.1016/j.tips.2023.04.006

[18] Pallotta MT, Orabona C, Volpi C, Vacca C, Belladonna ML, Bianchi R, Servillo G, Brunacci C, Calvitti M, Bicciato S, et al. Indoleamine 2,3-dioxygenase is a signaling protein in long-term tolerance by dendritic cells. Nat Immunol. 2011;12(9):870–878. 21804557 10.1038/ni.2077

[19] Shi Y, Hu G, Su J, Li W, Chen Q, Shou P, Xu C, Chen X, Huang Y, Zhu Z, et al. Mesenchymal stem cells: A new strategy for immunosuppression and tissue repair. Cell Res. 2010;20(5):510–518. 20368733 10.1038/cr.2010.44

[20] Gong C, Chang L, Sun X, Qi Y, Huang R, Chen K, Wang B, Kang L, Wang L, Xu B. Infusion of two-dose mesenchymal stem cells is more effective than a single dose in a dilated cardiomyopathy rat model by upregulating indoleamine 2,3-dioxygenase expression. Stem Cell Res Ther. 2022;13(1):409. 35962420 10.1186/s13287-022-03101-w PMC9373305

[21] Krampera M, Galipeau J, Shi Y, Tarte K, Sensebe L, MSC Committee of the International Society for Cellular Therapy (ISCT). Immunological characterization of multipotent mesenchymal stromal cells—The International Society for Cellular Therapy (ISCT) working proposal. Cytotherapy. 2013;15(9):1054–1061. 23602578 10.1016/j.jcyt.2013.02.010

[22] Tait Wojno ED, Hunter CA, Stumhofer JS. The immunobiology of the interleukin-12 family: Room for discovery. Immunity. 2019;50(4):851–870. 30995503 10.1016/j.immuni.2019.03.011 PMC6472917

[23] Jung JY, Gleave Parson M, Kraft JD, Lyda L, Kobe B, Davis C, Robinson J, Peña MM, Robinson CM. Elevated interleukin-27 levels in human neonatal macrophages regulate indoleamine dioxygenase in a STAT-1 and STAT-3-dependent manner. Immunology. 2016;149(1):35–47. 27238498 10.1111/imm.12625 PMC4981608

[24] Awasthi A, Carrier Y, Peron JP, Bettelli E, Kamanaka M, Flavell RA, Kuchroo VK, Oukka M, Weiner HL. A dominant function for interleukin 27 in generating interleukin 10-producing anti-inflammatory T cells. Nat Immunol. 2007;8(12):1380–1389. 17994022 10.1038/ni1541

[25] Campesato LF, Budhu S, Tchaicha J, Weng CH, Gigoux M, Cohen IJ, Redmond D, Mangarin L, Pourpe S, Liu C, et al. Blockade of the AHR restricts a Treg-macrophage suppressive axis induced by L-kynurenine. Nat Commun. 2020;11(1):4011. 32782249 10.1038/s41467-020-17750-z PMC7419300

[26] Rolvering C, Zimmer AD, Ginolhac A, Margue C, Kirchmeyer M, Servais F, Hermanns HM, Hergovits S, Nazarov PV, Nicot N, et al. The PD-L1- and IL6-mediated dampening of the IL27/STAT1 anticancer responses are prevented by α-PD-L1 or α-IL6 antibodies. J Leukoc Biol. 2018;104(5):969–985. 30040142 10.1002/JLB.MA1217-495R

[27] Carbotti G, Barisione G, Airoldi I, Mezzanzanica D, Bagnoli M, Ferrero S, Petretto A, Fabbi M, Ferrini S. IL-27 induces the expression of IDO and PD-L1 in human cancer cells. Oncotarget. 2015;6(41):43267–43280. 26657115 10.18632/oncotarget.6530 PMC4791231

[28] Lee SK, Silva DG, Martin JL, Pratama A, Hu X, Chang PP, Walters G, Vinuesa CG. Interferon-γ excess leads to pathogenic accumulation of follicular helper T cells and germinal centers. Immunity. 2012;37(5):880–892. 23159227 10.1016/j.immuni.2012.10.010

[29] Sugiyama N, Nakashima H, Yoshimura T, Sadanaga A, Shimizu S, Masutani K, Igawa T, Akahoshi M, Miyake K, Takeda A, et al. Amelioration of human lupus-like phenotypes in MRL/*lpr* mice by overexpression of interleukin 27 receptor α (WSX-1). Ann Rheum Dis. 2008;67(10):1461–1467. 18094002 10.1136/ard.2007.077537 PMC2566534

[30] Frigault MJ, Yao N, Berger TR, Wehrli M, Gallagher KME, Horick N, Graham CE, Jacobson CA, Chen YB, Leick MB, et al. Single-cell dynamics of breakthrough toxicities after anakinra prophylaxis for axicabtagene ciloleucel in lymphoma. Blood Adv. 2025;9(9):2122–2135. 39928957 10.1182/bloodadvances.2024015161 PMC12051123

[31] Meisel R, Zibert A, Laryea M, Göbel U, Däubener W, Dilloo D. Human bone marrow stromal cells inhibit allogeneic T-cell responses by indoleamine 2,3-dioxygenase-mediated tryptophan degradation. Blood. 2004;103(12):4619–4621. 15001472 10.1182/blood-2003-11-3909

[32] François M, Romieu-Mourez R, Li M, Galipeau J. Human MSC suppression correlates with cytokine induction of indoleamine 2,3-dioxygenase and bystander M2 macrophage differentiation. Mol Ther. 2012;20(1):187–195.21934657 10.1038/mt.2011.189

[33] Dominici M, Le Blanc K, Mueller I, Slaper-Cortenbach I, Marini F, Krause D, Deans R, Keating A, Prockop D, Horwitz E. Minimal criteria for defining multipotent mesenchymal stromal cells. The International Society for Cellular Therapy position statement. Cytotherapy. 2006;8(4):315–317. 16923606 10.1080/14653240600855905

[34] Zhou C, Bai X, Yang Y, Shi M, Bai XY. Single-cell sequencing informs that mesenchymal stem cell alleviates renal injury through regulating kidney regional immunity in lupus nephritis. Stem Cells Dev. 2023;32(15–16):465–483. 37082951 10.1089/scd.2023.0047

[35] Chen P, Tang S, Li M, Wang D, Chen C, Qiu Y, Fang Z, Zhang H, Gao H, Weng H, et al. Single-cell and spatial transcriptomics decodes Wharton’s jelly-derived mesenchymal stem cells heterogeneity and a subpopulation with wound repair signatures. Adv Sci. 2023;10(4): Article e2204786. 10.1002/advs.202204786 PMC9896049 36504438

[36] Guo Y, Lu X, Chen Y, Rendon B, Mitchell RA, Cuatrecasas M, Cortés M, Postigo A, Liu Y, Dean DC. Zeb1 induces immune checkpoints to form an immunosuppressive envelope around invading cancer cells. Sci Adv. 2021;7(21):eabd7455. 34020945 10.1126/sciadv.abd7455 PMC8139582

[37] Liu Y, Liu Y, Niu X, Chen A, Li Y, Yu Y, Mo B, Liu Z, Xu T, Cheng J, et al. Massively parallel interrogation of human functional variants modulating cancer immunosurveillance. Signal Transduct Target Ther. 2025;10(1):88. 40102418 10.1038/s41392-025-02171-5 PMC11920242

[38] Yan J, Chen D, Ye Z, Zhu X, Li X, Jiao H, Duan M, Zhang C, Cheng J, Xu L, et al. Molecular mechanisms and therapeutic significance of tryptophan metabolism and signaling in cancer. Mol Cancer. 2024;23(1):241. 39472902 10.1186/s12943-024-02164-y PMC11523861

[39] Mor A, Tankiewicz-Kwedlo A, Ciwun M, Lewkowicz J, Pawlak D. Kynurenines as a novel target for the treatment of inflammatory disorders. Cells. 2024;13(15):1259. 39120289 10.3390/cells13151259 PMC11311768

[40] Platten M, Nollen EAA, Röhrig UF, Fallarino F, Opitz CA. Tryptophan metabolism as a common therapeutic target in cancer, neurodegeneration and beyond. Nat Rev Drug Discov. 2019;18(5):379–401. 30760888 10.1038/s41573-019-0016-5

[41] Wang Y, Wu GR, Yue H, Zhou Q, Zhang L, He L, Gu W, Gao R, Dong L, Zhang H, et al. Kynurenine acts as a signaling molecule to attenuate pulmonary fibrosis by enhancing the AHR-PTEN axis. J Adv Res. 2024;71:521–532. 38906325 10.1016/j.jare.2024.06.017 PMC12126728

[42] Zhang Y, Tu S, Ji X, Wu J, Meng J, Gao J, Shao X, Shi S, Wang G, Qiu J, et al. *Dubosiella newyorkensis* modulates immune tolerance in colitis via the L-lysine-activated AhR-IDO1-Kyn pathway. Nat Commun. 2024;15(1):1333. 38351003 10.1038/s41467-024-45636-x PMC10864277

[43] Zuo M, Fang J, Huang P, Liu S, Hou P, Wang S, Liu Z, Feng C, Cao L, Li P, et al. IL4I1-catalyzed tryptophan metabolites mediate the anti-inflammatory function of cytokine-primed human muscle stem cells. Cell Death Discov. 2023;9(1):269. 37507432 10.1038/s41420-023-01568-x PMC10382538

[44] Li Y, Zhang D, Xu L, Dong L, Zheng J, Lin Y, Huang J, Zhang Y, Tao Y, Zang X, et al. Cell–cell contact with proinflammatory macrophages enhances the immunotherapeutic effect of mesenchymal stem cells in two abortion models. Cell Mol Immunol. 2019;16(12):908–920. 30778166 10.1038/s41423-019-0204-6 PMC6884632

[45] Wang G, Cao K, Liu K, Xue Y, Roberts AI, Li F, Han Y, Rabson AB, Wang Y, Shi Y. Kynurenic acid, an IDO metabolite, controls TSG-6-mediated immunosuppression of human mesenchymal stem cells. Cell Death Differ. 2018;25(7):1209–1223. 29238069 10.1038/s41418-017-0006-2 PMC6030103

[46] La Russa D, Di Santo C, Lizasoain I, Moraga A, Bagetta G, Amantea D. Tumor necrosis factor (TNF)-α-stimulated gene 6 (TSG-6): A promising immunomodulatory target in acute neurodegenerative diseases. Int J Mol Sci. 2023;24(2):1162. 36674674 10.3390/ijms24021162 PMC9865344

[47] Chen J, Yang H, Qin Y, Zhou X, Ma Q. Tryptophan ameliorates metabolic syndrome by inhibiting intestinal farnesoid X receptor signaling: The role of gut microbiota–bile acid crosstalk. Research. 2024;7: Article 0515. 39679283 10.34133/research.0515 PMC11638488

[48] Chen G, Ye Y, Cheng M, Tao Y, Zhang K, Huang Q, Deng J, Yao D, Lu C, Huang Y. Quercetin combined with human umbilical cord mesenchymal stem cells regulated tumour necrosis factor-α/interferon-γ-stimulated peripheral blood mononuclear cells *via* activation of Toll-like receptor 3 signalling. Front Pharmacol. 2020;11:499. 32390844 10.3389/fphar.2020.00499 PMC7194129

[49] Wu KK. Control of mesenchymal stromal cell senescence by tryptophan metabolites. Int J Mol Sci. 2021;22(2):697. 33445766 10.3390/ijms22020697 PMC7828284

[50] Gagliardi F, De Domenico P, Snider S, Roncelli F, Comai S, Mortini P. Immunomodulatory mechanisms driving tumor escape in glioblastoma: The central role of IDO and tryptophan metabolism in local and systemic immunotolerance. Crit Rev Oncol Hematol. 2025;209: Article 104657. 39986404 10.1016/j.critrevonc.2025.104657

[51] Villarino AV, Huang E, Hunter CA. Understanding the pro- and anti-inflammatory properties of IL-27. J Immunol. 2004;173(2):715–720. 15240655 10.4049/jimmunol.173.2.715

[52] Yang J, Chen X, Wu Y, Xu G, Qu X. Oncostatin M promotes osteogenic differentiation of tendon-derived stem cells through the JAK2/STAT3 signalling pathway. J Orthop Surg Res. 2024;19(1):407. 39014435 10.1186/s13018-024-04915-5 PMC11253339

[53] Brandstoetter T, Schmoellerl J, Grausenburger R, Kollmann S, Doma E, Huuhtanen J, Klampfl T, Eder T, Grebien F, Hoermann G, et al. SBNO2 is a critical mediator of STAT3-driven hematological malignancies. Blood. 2023;141(15):1831–1845. 36630607 10.1182/blood.2022018494 PMC10646773

[54] Dieudonne FX, Sévère N, Biosse-Duplan M, Weng JJ, Su Y, Marie PJ. Promotion of osteoblast differentiation in mesenchymal cells through Cbl-mediated control of STAT5 activity. Stem Cells. 2013;31(7):1340–1349. 23533197 10.1002/stem.1380

[55] Jiang Y, Glasstetter LM, Lerman A, Lerman LO. TSG-6 (tumor necrosis factor-α-stimulated gene/protein-6): An emerging remedy for renal inflammation. Hypertension. 2023;80(1):35–42. 36367104 10.1161/HYPERTENSIONAHA.122.19431 PMC9742181

[56] Lee SY, Moon SJ, Moon YM, Seo HB, Ryu JG, Lee AR, Lee CR, Kim DS, Her YM, Choi JW, et al. A novel cytokine consisting of the p40 and EBI3 subunits suppresses experimental autoimmune arthritis via reciprocal regulation of Th17 and Treg cells. Cell Mol Immunol. 2022;19(1):79–91. 34782759 10.1038/s41423-021-00798-2 PMC8752814

[57] Shi C, Zhang J, Wang H, Chen C, Han M, Gao L, Tang C, Sun P, Zhao X, Guo F, et al. Trojan horse nanocapsule enabled in situ modulation of the phenotypic conversion of Th17 cells to Treg cells for the treatment of multiple sclerosis in mice. Adv Mater. 2023;35(11): Article e2210262. 36575563 10.1002/adma.202210262

[58] Zhang Y, Jalili RB, Kilani RT, Elizei SS, Farrokhi A, Khosravi-Maharlooei M, Warnock GL, Ao Z, Marzban L, Ghahary A. IDO-expressing fibroblasts protect islet beta cells from immunological attack and reverse hyperglycemia in non-obese diabetic mice. J Cell Physiol. 2016;231(9):1964–1973. 26743772 10.1002/jcp.25301

[59] Kuca-Warnawin E, Skalska U, Janicka I, Musiałowicz U, Bonek K, Głuszko P, Szczęsny P, Olesińska M, Kontny E. The phenotype and secretory activity of adipose-derived mesenchymal stem cells (ASCs) of patients with rheumatic diseases. Cells. 2019;8(12):1659. 31861245 10.3390/cells8121659 PMC6952982

