# Supplementary material for: IL-27 Modulates Mesenchymal Stem Cell Immunoplasticity for Enhanced Lupus Nephritis Therapy via the JAK1–STAT1–IDO Axis and Tryptophan Metabolic Orchestration
Source: Research (Wash D C). 2025 Jul 10;8:0748. doi: 10.34133/research.0748 (PMC12241797; doi:10.34133/research.0748)
Supplement: Supplementary 1 — Figs. S1 to S5 [file research.0748.f1.docx]

Supplementary Materials for

**IL-27 Modulates Mesenchymal Stem Cell Immunoplasticity for Enhanced Lupus Nephritis Therapy via JAK1-STAT1-IDO Axis and Tryptophan Metabolic Orchestration**

Cheng Zhou *et al.*

*Corresponding author. Email:xueyuan_bai@163.com

**This file includes:**

Supplementary

Figs. S1 to S5


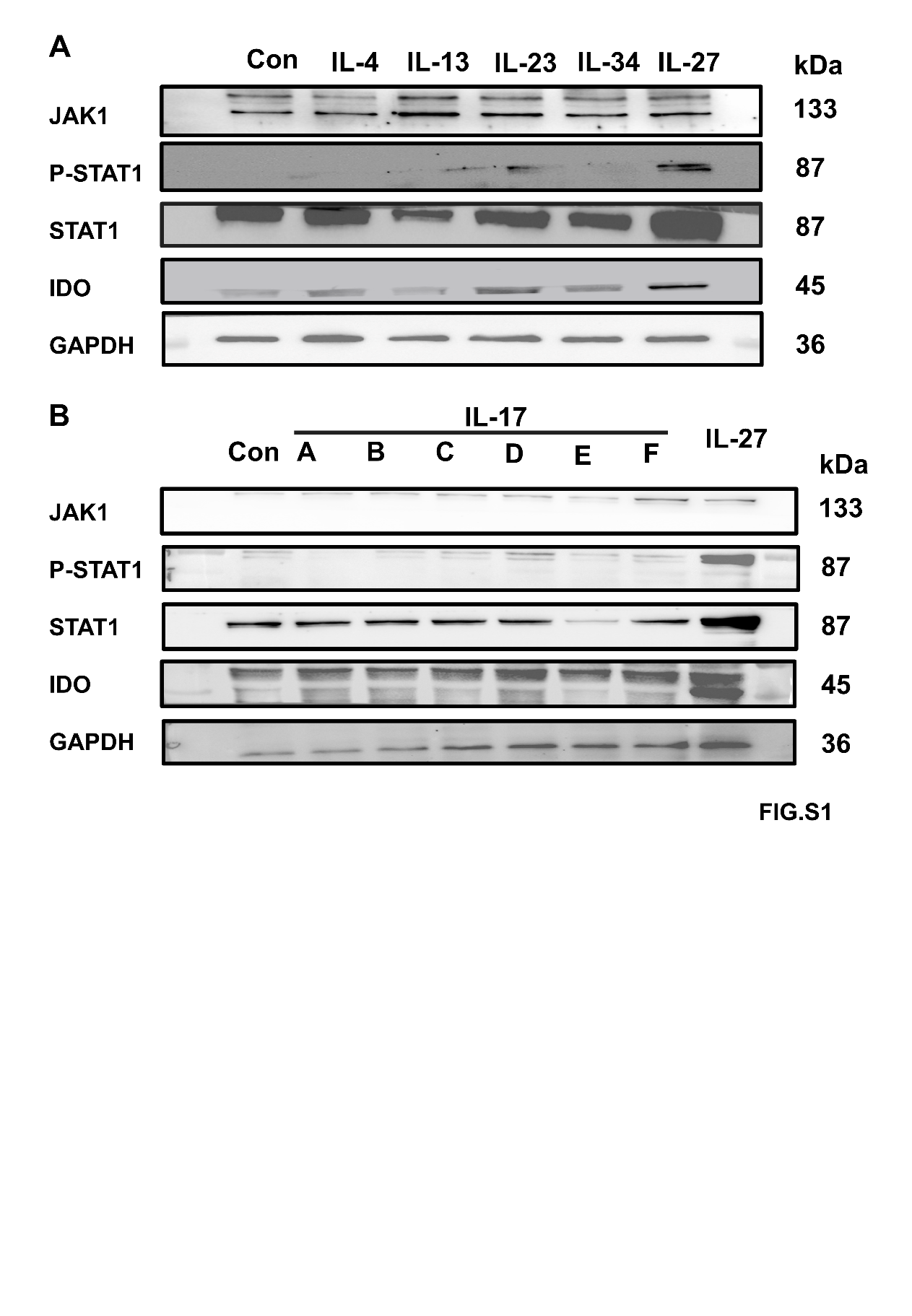


Fig. S1.

**Other cytokines had no influence on the protein expression of IDO in MSC in vitro.** **(A)** Western blot of protein expression of IDO in the MSC treated by different cytokines, such as, IL-4, IL-13, IL-33, IL-34. **(B)** Western blot of protein expression of IDO in the MSC treated by different cytokines, such as, IL-17A, IL-17B, IL-17C, IL-17D, IL-17E, IL-17F.


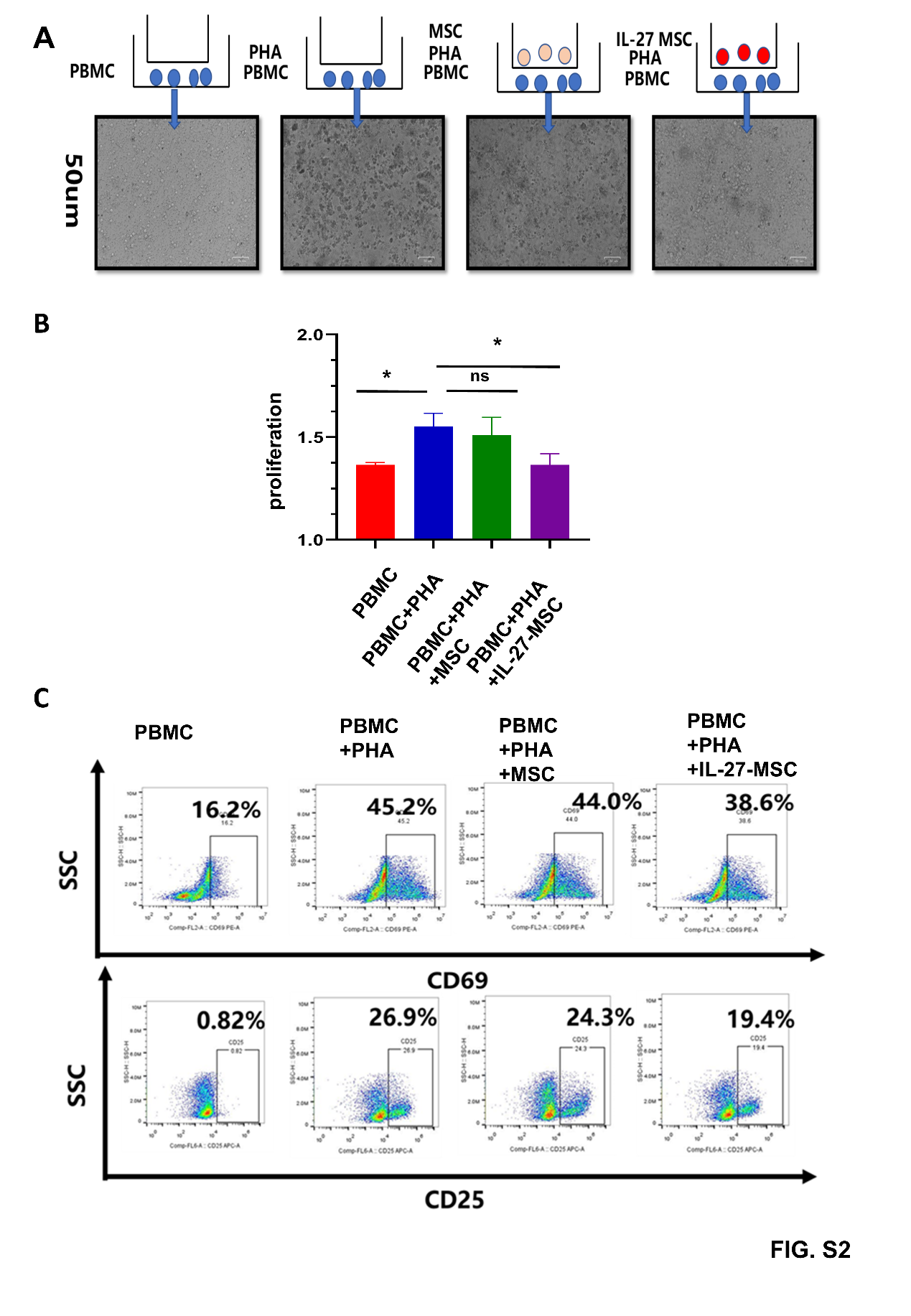


Fig. S2.

**IL-27 enhanced MSC immunosuppressive function on human PBMC in vitro.** **(A)** The representative picture of PBMC proliferation when the ratio of MSC to PBMC co-culture was 1:80. **(B)** The statistical histogram (right) of PBMC proliferation in different culturing groups. **(C)** Representative flow cytometry analysis of T-cell activation marker CD69 and CD25 in different culturing groups.


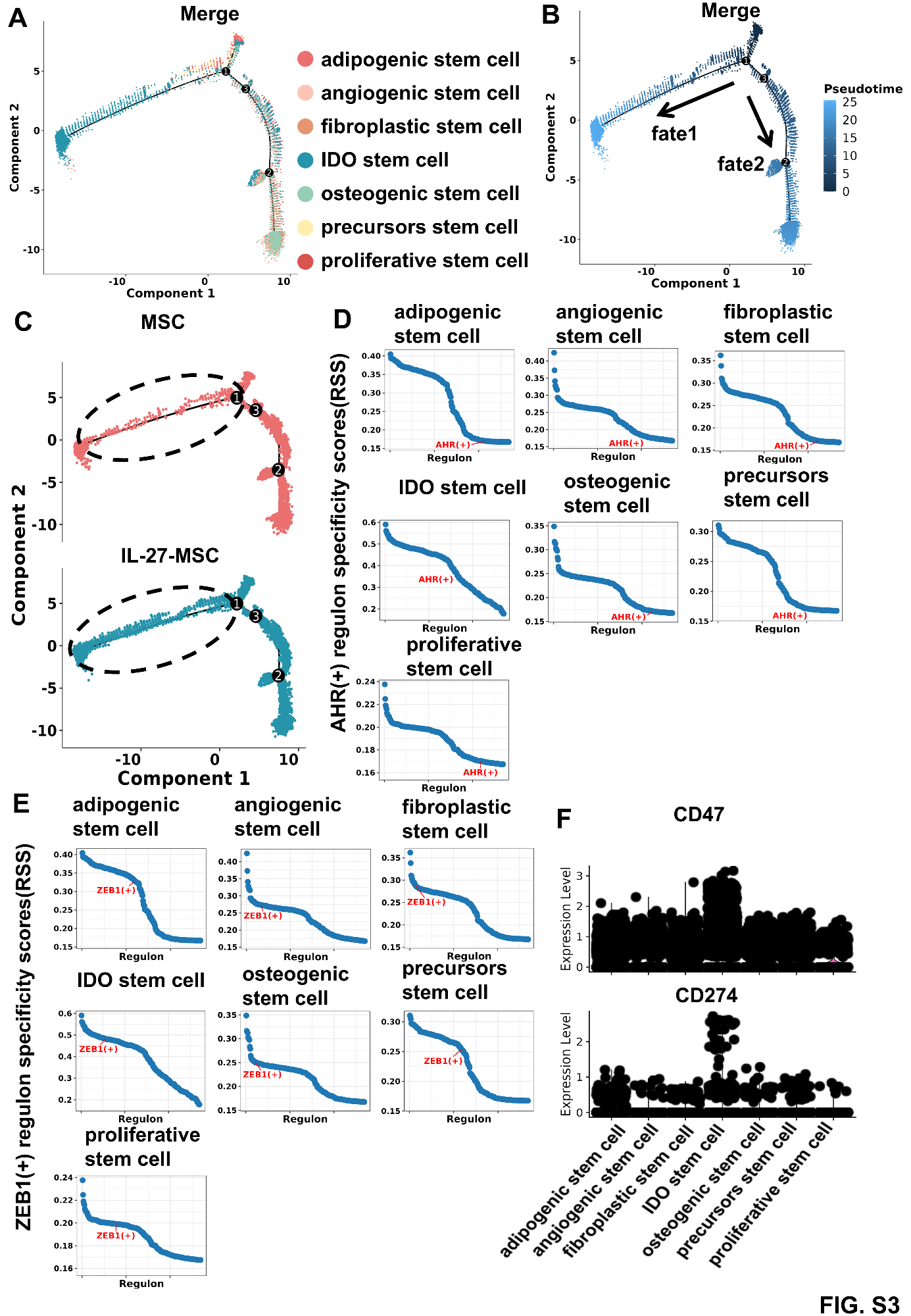


Fig. S3.

**Track of differentiation and transcription factor of 7 clusters in IDO+ subgroup in the merge groups (MSC and IL-27-MSC).** **(A)** Track of differentiation of 7 different clusters. **(B)** Pseudotemporal ordering trajectory map. Dark to light colors represent the pseudotime order. **(C)** Track of differentiation of MSC and IL-27-MSC groups. **(D)** AHR (+) regulon specificity scores of 7 different clusters. **(E)** ZEB1(+) regulon specificity scores of 7 different clusters. **(F)**Violin plot of CD47, CD274 genes elative expression levels of 7 different clusters.


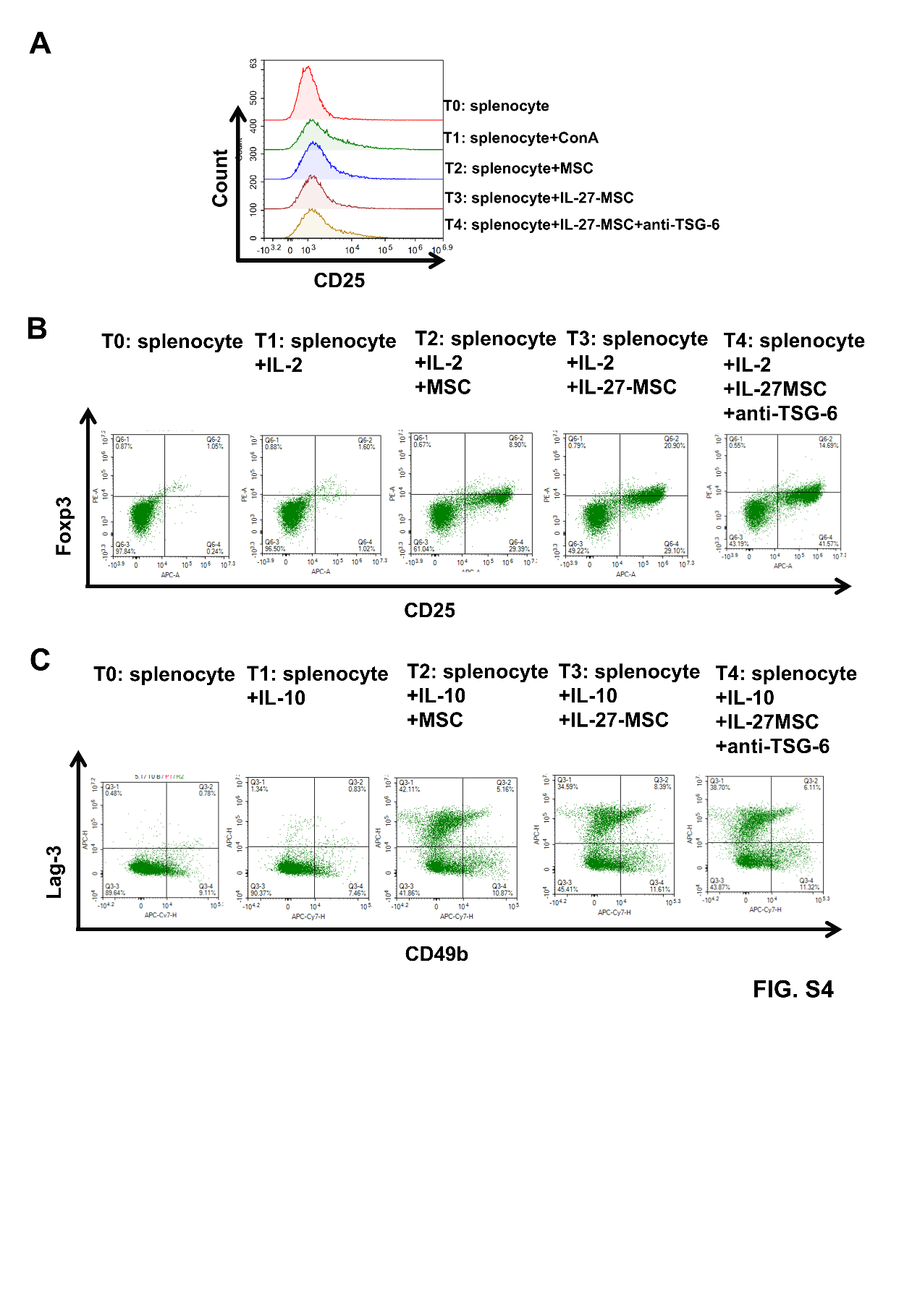


Fig. S4.

**Blocking TSG-6 reversed MSC immunosuppressive function by IL-27 in vitro.** **(A)** Representative flow cytometry analysis of T-cell activation marker CD25 in different culturing groups. **(B)** Representative flow cytometry analysis of CD25+Foxp3+Treg cell in different culturing groups. **(C)** Representative flow cytometry analysis of CD49b+Lag-3+Tr1 cell in different culturing groups.


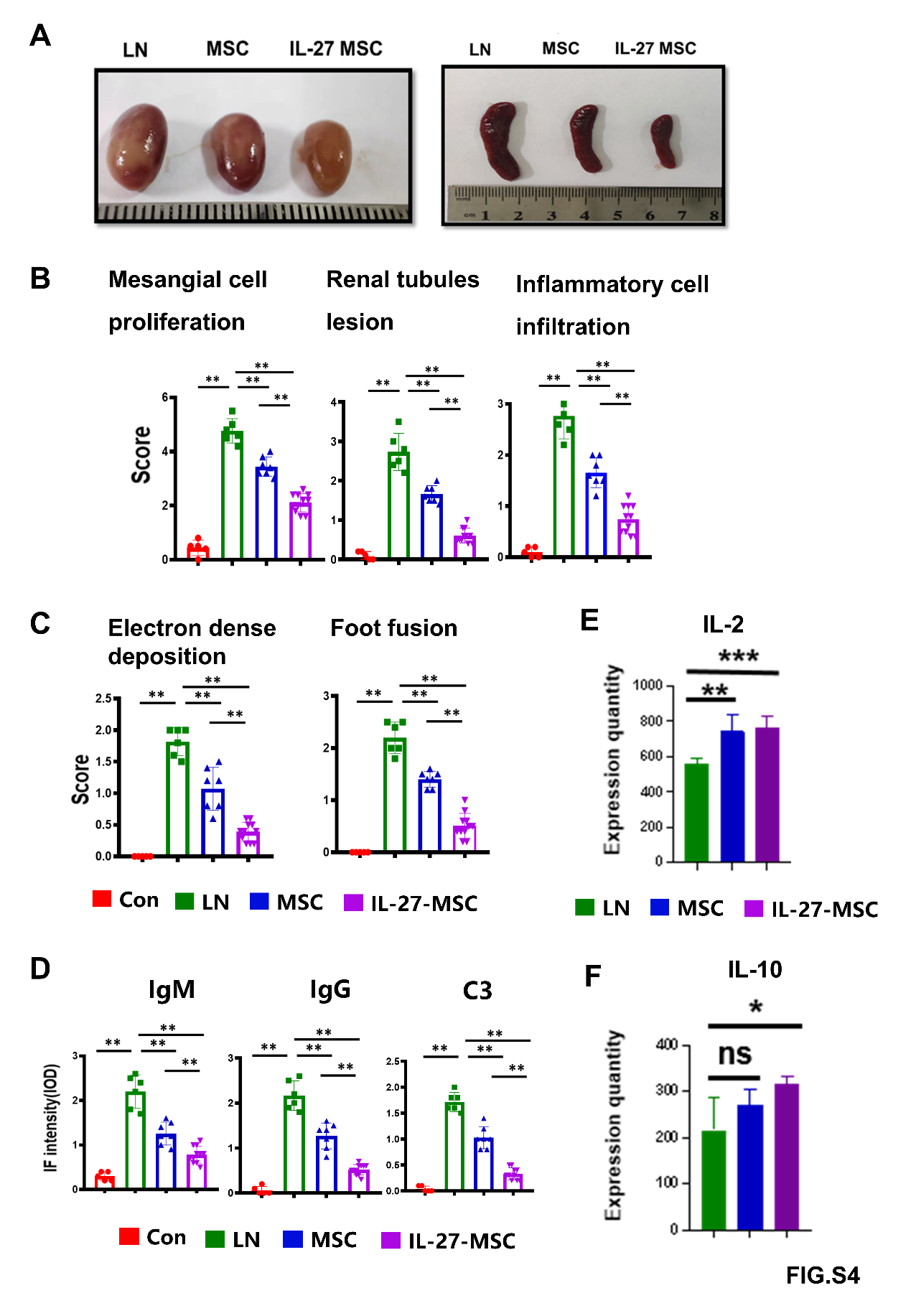


Fig. S5.

**IL-27 promotes therapeutic effects of MSC in renal pathological injury, deposition of IgM, IgG, C3 in renal tissues.** **(A)** The size changes of kidney and spleen in the LN, MSC and IL-27-MSC three groups. **(B)** Semiquantitative pathological score by PAS and HE (the score of mesangial cell proliferation, tubular lesion and inflammatory cell infiltration) in Con, LN, MSC, IL-27-MSC groups. **(C)** Semiquantitative pathological score by Electron microscope (the score of electron dense deposits and podocyte effacement) in Con, LN, MSC, IL-27-MSC groups. **(D)** Semiquantitative fluorescence intensity (IgM, IgG, and C3) in Con, LN, MSC, IL-27-MSC groups. Data from Con group(n=5), LN group(n=6), MSC group(n=7) and IL-27-MSC(n=10) are expressed as mean± SD. **(E, F).** Histogram of IL-2 and IL-10 expression in the LN, MSC and IL-27-MSC three groups by RayBiotech antibody chip(n=5/group). Data obtained from each group were compared using a one-way analysis of variance (ANOVA) followed by post hoc Tukey's multiple comparison test. *p<0.05, **p<0.01.
